# A Novel Acyl-AcpM-Binding Protein Confers Intrinsic Sensitivity to Fatty Acid Synthase Type II Inhibitors in *Mycobacterium smegmatis*

**DOI:** 10.3389/fmicb.2022.846722

**Published:** 2022-04-04

**Authors:** Mengmiao Li, Qian Huang, Weidi Zhang, Yinghua Cao, Zhanxin Wang, Zhenwen Zhao, Xiaotian Zhang, Junjie Zhang

**Affiliations:** ^1^Key Laboratory of Cell Proliferation and Regulation Biology, Ministry of Education, Department of Biology, College of Life Sciences, Beijing Normal University, Beijing, China; ^2^Key Laboratory of Analytical Chemistry for Living Biosystems, Institute of Chemistry, Chinese Academy of Sciences, Beijing, China

**Keywords:** MSMEG_5634, acyl-AcpM, FAS-II inhibitor, *Mycobacterium smegmatis*, Rv0910

## Abstract

The fatty acid synthase type II (FAS-II) multienzyme system is the main target of drugs to inhibit mycolic acid synthesis in mycobacterium. Meromycolate extension acyl carrier protein (AcpM) serves as the carrier of fatty acyl chain shuttling among the individual FAS-II components during the progression of fatty acid elongation. In this paper, MSMEG_5634 in *Mycobacterium smegmatis* was determined to be a helix-grip structure protein with a deep hydrophobic pocket, preferring to form a complex with acyl-AcpM containing a fatty acyl chain at the C36-52 length, which is the medium product of FAS-II. MSMEG_5634 interacted with FAS-II components and presented relative accumulation at the cellular pole. By forming the MSMEG_5634/acyl-AcpM complex, which is free from FAS-II, MSMEG_5634 could transport acyl-AcpM away from FAS-II. Deletion of the *MSMEG_5634* gene in *M. smegmatis* resulted in a mutant with decreased sensitivity to isoniazid and triclosan, two inhibitors of the FAS-II system. The isoniazid and triclosan sensitivity of this mutant could be restored by the ectopic expression of MSMEG_5634 or Rv0910, the MSMEG_5634 homologous protein in *Mycobacterium tuberculosis* H37Rv. These results suggest that MSMEG_5634 and its homologous proteins, forming a novel acyl-AcpM-binding protein family in mycobacterium, confer intrinsic sensitivity to FAS-II inhibitors.

## Introduction

Mycolic acids are long-chain α-hydroxy β-alkyl fatty acids with two chains: a short alkyl chain called the α branch and a long meroaldehyde chain called the meromycolic acid (meromycolate) chain (Marrakchi et al., 2014). Mycolic acids are essential components of the mycobacterial cell wall that play roles in cell viability, virulence, and drug tolerance (Gurvitz et al., 2008; Guenin-Mace et al., 2009). In mycobacteria, mycolic acids are synthesized through two fatty acid elongation systems, fatty acid synthase type I (FAS-I), and fatty acid synthase type II (FAS-II). FAS-I is a multifunctional polypeptide containing all the functional domains required for *de novo* fatty acid synthesis to produce saturated α branches, while FAS-II is composed of discrete enzymes to produce meromycolate chains (PaweLczyk and Kremer, 2014). The processes of meromycolate elongation by FAS-II mainly occur in three distinct steps. First, FabD transfers the malonate moiety from malonyl-CoA to acyl carrier protein (ACP) to form malonyl-ACP (Kremer et al., 2001), and FabH condenses acyl-CoA (generated from FAS-I) with malonyl-ACP to form β-ketoacyl-ACP (Bhatt et al., 2007). Second, the elongation process is performed through an iterative series of reactions, which are catalyzed by the turn of β-ketoacyl-ACP reductase MabA (Marrakchi et al., 2002), β-hydroxyacyl-ACP dehydratases HadABC (Sacco et al., 2007), trans-2-enoyl-ACP reductase InhA (Banerjee et al., 1994), and β-ketoacyl ACP synthases KasA/KasB (Slayden and Barry, 2002), to add a two-carbon unit of a nascent acyl group per reaction cycle until the meromycolate chains reach C42–62 in length. Finally, the α branch chains generated from FAS-I and the meromycolate chains generated from FAS-II are condensed by polyketide synthase 13 (Pks13) to produce mycolic acids (Gande et al., 2004). The FAS-II complex with its component enzymes displays polar localization, as mycolic acids are mainly synthesized at the growing pole of mycobacteria (Carel et al., 2014). The process of mycolic acid synthesis, especially the FAS-II system, is the target of anti-tuberculosis drugs. InhA and KasA are regarded as the main targets of the well-known anti-tuberculosis drug isoniazid (INH) (Slayden and Barry, 2002; Vilcheze and Jacobs, 2014; Unissa et al., 2016). Triclosan (TRC) is a chemical drug developed recently with InhA as its target (Parikh et al., 2000).

In mycobacteria, meromycolate extension acyl carrier protein (AcpM) plays a central role in sequestering and transporting fatty acyl chains among the FAS-II enzymes (Marrakchi et al., 2014). AcpM is posttranslationally modified by a phosphopantetheine arm, which is subsequently covalently linked with the fatty acyl chain to form acyl-AcpM (Wong et al., 2002). When acyl-AcpM binds with different FAS-II enzymes, the fatty acyl chain of acyl-AcpM is translocated from its hydrophobic core and inserts into the catalytic center of FAS-II enzymes. With transient and reversible interactions, acyl-AcpM shuttles among the individual FAS-II components during fatty acid elongation (Rock and Jackowski, 2002).

In this study, MSMEG_5634 in *Mycobacterium smegmatis* was identified as a novel acyl-AcpM-binding protein. MSMEG_5634 was found to have a helix-grip structure with a deep hydrophobic pocket, preferring to interact with acyl-AcpM containing a fatty acyl chain at the C36‑52 length. MSMEG_5634 could transport acyl-AcpM away from FAS-II by forming an MSMEG_5634/acyl-AcpM complex. Deletion of the *MSMEG_5634* gene in *M. smegmatis* resulted in a mutant with tolerance to isoniazid and triclosan, two FAS-II inhibitors. The isoniazid sensitivity and triclosan sensitivity of this mutant were restored when it was complemented with the expression of MSMEG_5634 or its homologous protein Rv0910 from *Mycobacterium tuberculosis*. The biological functions of MSMEG_5634 and its homologous proteins are further discussed in this paper.

## Materials and Methods

### Strains and Media

*Escherichia coli* BL21 (DE3) and *E. coli* B834 (DE3) were cultured in Luria-Bertani broth at 37°C. *Mycobacterium smegmatis* mc^2^155 was cultured in Middlebrook 7H9 broth (BD, New York, United States) supplemented with 0.2% (v/v) glycerol, 0.05% (v/v) Tween-80, and OADC (0.006% oleic acid, 0.5% bovine serum albumin, 0.2% dextrose, 0.0003% catalase, and 0.085% sodium chloride) at 37°C or Middlebrook 7H10 solid medium (BD, New York, United States) supplemented with 0.5% (v/v) glycerol and OADC at 37°C. The *MSMEG_5634* gene deletion (∆5634) strain was constructed by homologous recombination with p1NIL and pGOAL19 vectors according to the protocol described previously (Parish and Stoker, 2000; Kendall and Frita, 2009) as described in the supplementary data (Supplementary Figure S10). The final concentrations of antibiotics were as follows: 100 μg/ml ampicillin or 50 μg/ml kanamycin for *E. coli*, 50 μg/ml hygromycin or 25 μg/ml kanamycin for *M. smegmatis*.

### Vector Construction

The *MSMEG_5634* gene was inserted into a modified His_6_-SUMO-tagged pRSFDuet-1 vector between Nde I and Not I restriction sites to create a plasmid expressing His_6_-SUMO-tagged MSMEG_5634 protein in *E. coli*. To create the plasmid to express MSMEG_5634 with a C-terminal Flag tag or Rv2801c with a C-terminal His_6_ tag in *E. coli*, the corresponding gene was inserted into the pET21cc vector between the Nde I and Xho I restriction sites, respectively. To create the plasmid expressing MSMEG_5635 with an N-terminal His_6_ tag or Rv2801a with a N-terminal Flag tag in *E. coli*, the *MSMEG_5635* gene was inserted into the pET28a vector between the Nde I and Hind III restriction sites, and the gene encoding Flag-Rv2801a into the NcoI and Hind III restriction sites. For protein expression in *M. smegmatis*, the gene encoding MSMEG_5634 or its mutant with a C-terminal His_6_ tag was inserted into the pACE vector between BamH I and Cla I restriction sites, and the gene encoding AcpM with a C-terminal Flag tag was inserted into the pMV261 vector between BamH I and Hind III restriction sites. MSMEG_5634 fused with GFP at the C-terminus was expressed with the pACE vector to detect its cellular location in *M. smegmatis*.

### Protein Purification

*Escherichia coli* B834 (DE3) carrying the pRSFDuet-1 vector expressing His_6_-SUMO-tagged MSMEG_5634 protein was grown at 37°C until OD_600_ nm ≈ 1.0 and harvested by centrifugation at 4,000 rpm for 10 min. After washing with distilled water, the bacteria were transferred into seleno-nutrient mixed medium with 50 mg/L selenomethionine, and then, IPTG (0.2 mM) was added to induce expression overnight at 20°C. The bacteria were collected, and the pellets were resuspended in lysis buffer (20 mM Tris, 500 mM NaCl, and 20 mM imidazole, pH 7.0) and sonicated for 10 min. The lysate was centrifuged at 18,000 × g for 30 min, and then, the supernatant was collected. The His_6_-SUMO-tagged MSMEG_5634 protein was isolated through a nickel-charged HiTrap Chelating FF column from GE Healthcare. The His_6_-SUMO tag was cleaved by incubating with histidine-tagged ULP1 protease and then dialyzed with lysis buffer at 4°C overnight. The dialyzed solution was then reloaded into a nickel-charged chelating column to remove both the histidine-tagged SUMO and ULP1. The protein samples were further purified by size exclusion chromatography on a Superdex^TM^ 200 PG, T 10/300 Gl.

### Protein Crystallization and Structure Analysis

Crystallization was carried out using the hanging-drop, vapor-diffusion method by mixing equal volumes of protein and well solution. Crystals of MSMEG_5634 were grown at 25°C by mixing 0.5 μl of protein at a concentration of 25 mg/ml with 0.5 μl crystallization buffer (0.2 M calcium acetate hydrate, 20% w/v polyethylene glycol 3,350, pH 7.5). Datasets for crystals of the selenomethionine-labeled protein samples were collected at the Shanghai Synchrotron Radiation Facility (SSRF) beamline BL17U1 in China at a wavelength of 0.97930 Å. All the datasets were processed by the program HKL2000 (Otwinowski and Minor, 1997). Structure determination was carried out by PHENIX (Adams et al., 2010) using the datasets of the selenomethionine-labeled crystals of protein samples through the SAD method. All of the selenium atoms were identified and used to solve the initial phase with a partial model. The partial model was manually rebuilt by Winn et al. (2011) and further refined by PHENIX (Murshudov et al., 1997; Adams et al., 2010; Winn et al., 2011). The coordinate of MSMEG_5634 has been deposited in the PDB with the accession number 7WA9.

### Pull-Down Assays

To perform pull-down assays in *E. coli*, *E. coli* cells carrying the indicated protein expression vectors constructed from pET21cc and pET28a were cultured at 37°C until OD_600_ nm ≈ 0.6, and then, 0.2 mM IPTG was added to induce protein expression for 4 h at 37°C. To perform pull-down assays in *M. smegmatis*, the indicated protein expression vectors constructed from pACE or pMV261 were electroporated into *M. smegmatis*. *M. smegmatis* cells carrying the vectors were cultured at 37°C until OD_600_ nm ≈ 1.0, and then, 0.2% acetamide was added to induce expression for 8 h at 37°C. The bacteria were centrifuged at 4,000 rpm for 10 min for collection. The pellets were resuspended in lysis buffer (50 mM Tris, 150 mM NaCl, and 10 mM imidazole, pH 7.0) and then sonicated for approximately 10 min (*E. coli*) or 20 min (*M. smegmatis*). The lysate was centrifuged at 13,000 × g for 30 min, and the supernatant was collected. The His_6_-tagged proteins were pulled down by Ni^2+^-NTA resin (Novagen, Malaysia) according to the standard procedure. The supernatant was incubated with Ni^2+^-NTA resin at 4°C for 4 h. The resin was collected and washed three times with wash buffer (50 mM Tris, 150 mM NaCl, and 20 mM imidazole, pH 7.0). The binding proteins were eluted with elution buffer (50 mM Tris, 150 mM NaCl, and 250 mM imidazole, pH 7.0). The Flag-tagged proteins were purified with FLAG Affinity Gels (Macgene, Beijing, China) according to the standard procedure. The supernatant was incubated with FLAG Affinity Gels at 4°C for 4 h. The precipitated protein samples were collected and analyzed by Tricine-SDS-PAGE and Western blot.

### Tricine-SDS-PAGE and Western Blot

Tricine-SDS-PAGE was prepared according to the protocol described previously (Schagger, 2006). The Tricine-SDS-PAGE gels consisted of a stacking layer, a spacer layer, and a separating layer. The stacking layer contained 1 M Tris·HCl (pH 8.45), 0.1% (wt/vol) SDS, and 4% (wt/vol) acrylamide/bis (29:1) solution. The stacking layer contained 1 M Tris·HCl (pH 8.45), 0.1% (wt/vol) SDS, 10% (vol/vol) glycerol, and 10% (wt/vol) acrylamide/bis (29:1) solution. The separating layer contained 1 M Tris·HCl (pH 8.45), 0.1% (wt/vol) SDS, 10% (vol/vol) glycerol, 6 M urea, and 16% (wt/vol) acrylamide/bis (29:1) solution. The anode running buffer consisted of 0.1 M Tris·HCl (pH 8.9), and the cathode running buffer consisted of 0.1 M Tris·HCl, 0.1% (wt/vol) SDS, and 0.1 M Tricine (pH 8.9). Anti-Flag and anti-His_6_ tag antibodies were purchased from MBL (MBL Beijing Biotech, Beijing, China). For immunoblotting analysis, protein samples were loaded into Tricine-SDS-PAGE for electrophoretic separation, transferred to PVDF membrane, and then subjected to immunodetection using standard procedures.

### Mass Spectrometry and Peptide Mapping

To identify proteins interacting with MSMEG_5634, protein bands were excised from Tricine-SDS-PAGE gel and digested with trypsin. Peptide mixture was mixed with matrix α-cyano-4-hydroxycinnamic acid (CHCA) after desalting with C18ZipTip, and samples were analyzed by MALDI-TOF/TOF Ultraflextreme^TM^ (Brucker, Germany). Data were collected and searched through matrixscience.^1^

To identify proteins copurified with MSMEG_5634, protein bands were excised from a Tricine-SDS-PAGE gel and digested with trypsin. After desalting with C18 ZipTip, we performed UPLC-ESI-HR-MS analysis by ultimate 3,000 liquid chromatography coupled to an LTQ Orbitrap mass spectrometer. Liquid chromatographic separations of the analyses were performed using a Thermo Hypersil BDS C18 column (75 μm × 15 cm, 3 μm). The mobile phase consisted of 0.1% formic acid in water (solvent A), 0.1% formic acid in 80% acetonitrile, and 20% water (solvent B). The gradient elution was optimized as follows: 0–3 min, linear from 95% to 87% A; 3–81 min, linear from 87% to 55% A; 81–83 min, linear from 55% to 0% A; and 83–90 min, held at 0% A. The injection volume was 2 μl. The flow rate was 300 nl/min. The temperature-controlled column oven was set at 30°C, and the sample was set at 4°C. The ESI source was operated in the positive mode. Full MS scans were acquired in the range m/z 300–1,600 with a mass resolution of 30,000. Proteome Discoverer (version 1.4.0.288, Thermo Fisher Scientific) was used for data collection and analysis. Data were searched through UniProt_ proteome_ *Mycobacterium*_ *smegmatis*_ 2018. To determine the molecular weights of MSMEG_5634-His_6_ and acyl-AcpM, the purified MSMEG_5634-His_6_/acyl-AcpM complex was subjected to UPLC-ESI-HR-MS as described above.

### Scanning Electron Microscopy

The *M. smegmatis* wild-type strain and the *MSMEG_5634* gene deletion (∆5634) strain were grown to logarithmic phase (OD_600_ nm = 1.0) in 7H9 medium and then treated with 12.5 μg/ml for 12 h. The cell pellets were washed three times with 0.1 M phosphate buffer and then incubated with 2.5% (vol/vol) glutaraldehyde in 0.1 M phosphate buffer (pH 7.4) at 4°C overnight. The cells were dehydrated in a graded series of 30%, 50%, 75%, 80%, 90%, and 100% ethanol, dried by critical point drying with CO_2_, and coated with gold. The cells were observed using a JEOL JSM-6701f scanning electron microscopy.

### Minimum Inhibitory Concentration

The susceptibility of different strains to isoniazid or triclosan was determined on 96-well cell culture plates. Briefly, approximately 10^5^ cells/well were incubated for 24 h with different drug concentrations at 37°C. The indicator 0.02% TTC (2,3,5-triphenyl-2H-tetrazolium chloride; Sangon, Shanghai, China) was then added to individual samples, and color changes (from white to red) were recorded after 4 h. White indicated no growth, and red indicated growth. The MIC was defined as the lowest antibiotic drug concentration that prevented the color change from white to red.

### Statistical Analysis

All experiments were independently repeated three times. The data are shown as the mean ± SD of *n* = 3 independent experiments.

## Results

### MSMEG_5634 Does Not Function as a Toxin Protein in the Toxin-Antitoxin System

**Figure 1:**
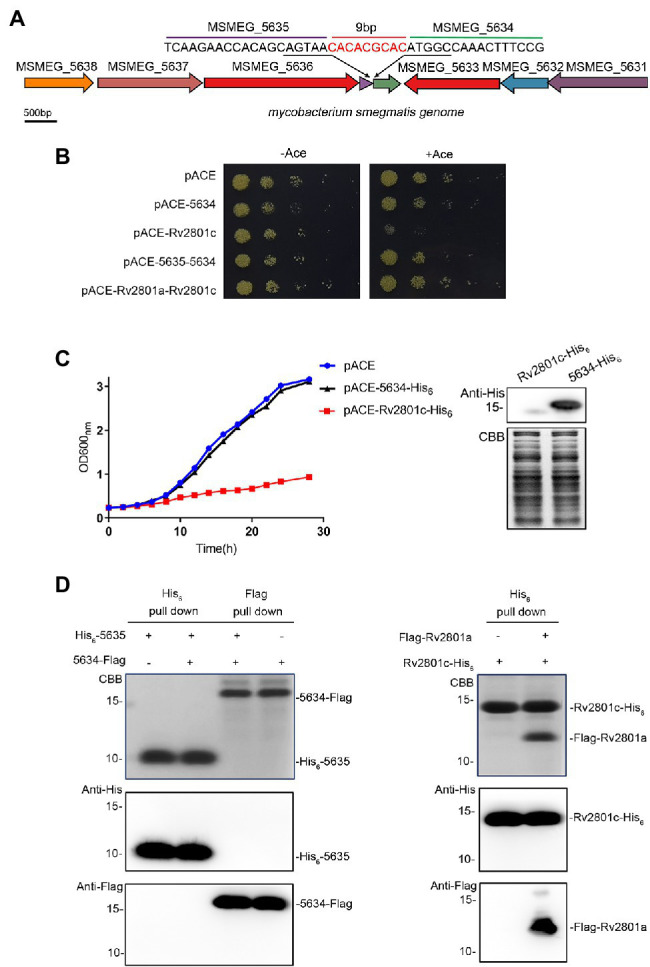
MSMEG_5634 is not a toxin protein in the toxin-antitoxin system. **(A)** MSMEG_5634 gene locus in the *Mycobacterium smegmatis* genome. **(B)** *M. smegmatis* cells expressing MSMEG_5634 alone or MSMEG_5634–5635 under the control of the inducible acetamidase promoter in the pACE vector were serially diluted and plated on 7H10 solid medium with (right panel) or without (left panel) 0.2% acetamide. *M. smegmatis* cells expressing Rv2801c alone or Rv2801a-Rv2801c with the pACE vector were used as controls. **(C)** *M. smegmatis* cells expressing MSMEG_5634-His_6_ under the control of the inducible acetamidase promoter in the pACE vector were cultured in 7H9 liquid medium with 0.2% acetamide. *M. smegmatis* cells harboring empty pACE vector and *M. smegmatis* cells expressing Rv2801c-His_6_ with the pACE vector were used as controls. The OD_600_ nm of each *M. smegmatis* culture was measured every 2 h (left panel). The expression levels of Rv2801c-His_6_ and MSMEG_5634-His_6_ were detected by Western blot (right panel). **(D)** The interaction between MSMEG_5634 and MSMEG_5635 was detected by pull-down assays. His_6_-tagged MSMEG_5635 and Flag-tagged MSMEG_5634 were coexpressed in *E. coli*. Flag-tagged MSMEG_5634 was purified by Flag affinity chromatography, His_6_-tagged MSMEG_5635 was purified by Ni^2+^NTA affinity chromatography, respectively, and then, the eluted proteins were analyzed by Tricine-SDS-PAGE and Western blot (left panel). The interactions between Rv2801c and Rv2801a were detected as a control (right panel).

*MSMEG_5634* (*MSMEG_RS27155*) is a hypothetical gene located downstream of *MSMEG_5635* in the *M. smegmatis* genome (Figure 1A). The RNA levels of *MSMEG_5634* and *MSMEG_5635* could be detected by RT-PCR (Supplementary Figure S1). *MSMEG_5635* and *MSMEG_5634* are expressed in a polycistronic operon with six upstream genes (Gupta et al., 2017). It has been reported that *MSMEG_5634* encodes a toxic protein whose expression leads to the growth arrest of *M. smegmatis* and forms a toxin-antitoxin system (TA system) together with *MSMEG_5635* (Ramage et al., 2009). However, when we detected the toxicity of MSMEG_5634 with the classical toxin protein Rv2801c as a control, it was found that the expression of MSMEG_5634 did not inhibit the growth of *M. smegmatis*, while the expression of Rv2801c was obviously toxic (Figures 1B,C; Supplementary Figures S2, S3). Another characteristic of the typical TA system is that each toxin protein is able to interact with its corresponding antitoxin protein to form a toxin-antitoxin complex. His_6_-tagged MSMEG_5634 and Flag-tagged MSMEG_5635 were coexpressed in *E. coli*, and then, the interaction between MSMEG_5634 and MSMEG_5635 was detected by pull-down assays. The results showed that MSMEG_5634 could not be copurified with MSMEG_5635. Meanwhile, regarding the known Rv2801a/Rv2801c TA system, toxin protein Rv2801c could be copurified with its antitoxin Rv2801a (Figure 1D). These data indicate that MSMEG_5634 is not a toxin protein in the TA system.

### Structure of the MSMEG_5634 Protein

**Figure 2:**
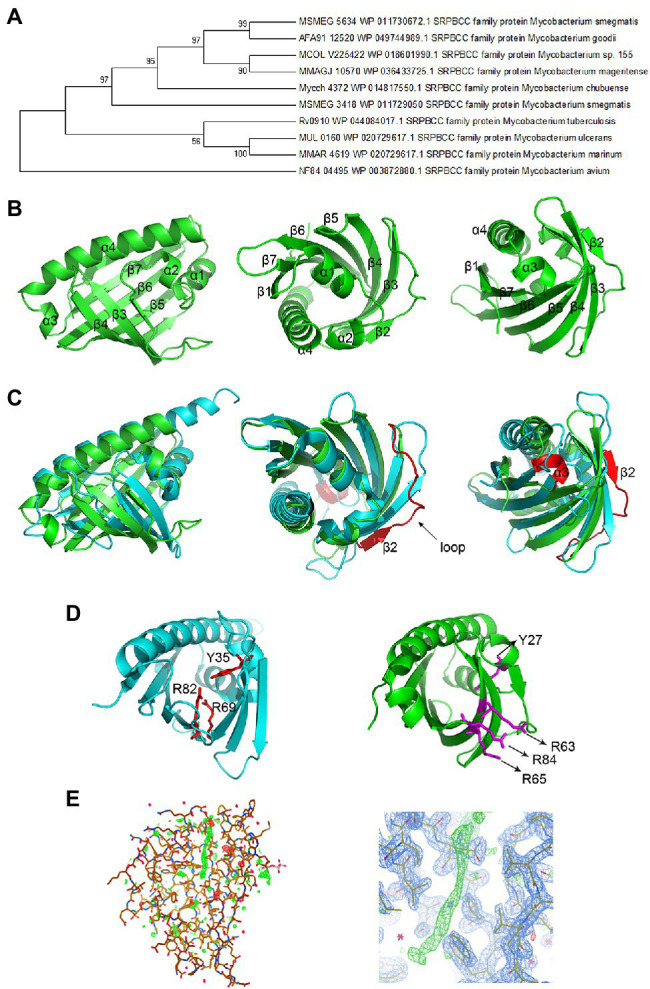
Structure of the MSMEG_5634 protein. **(A)** The evolutionary tree of the MSMEG_5634 protein. NCBI PSI-BLAST (Position-Specific Iterated BLAST) and KEGG BLAST were performed to search for homologs of MSMEG_5634. The evolutionary tree was constructed through MAGE 7.0. Larger taxonomic clusters have been collapsed for clarity. **(B)** The overall structure of MSMEG_5634. **(C)** The structural superimposition of MSMEG_5634 (green) and TcmN ARO/CYC (cyan) performed using PyMOL. The major differences between MSMEG_5634 and TcmN ARO/CYC are colored red. **(D)** The catalytic center of TcmN (cyan) and the corresponding sites in MSMEG_5634 (green). The cyclization or aromatization of polyketides is mediated by Tyr-35, Arg-69, and Arg-82 in TcmN ARO/CYC. The corresponding residues in MSMEG_5634 (green) are Tyr-27, Arg-63, Arg-65, and Arg-84. **(E)** The 2Fo-Fc electron density map of MSMEG_5634 contoured at 1 sigma level. A linear electron density was detected inserting into the hydrophobic pocket of MSMEG_5634.

NCBI PSI-BLAST and KEGG BLAST were performed to search the homologous proteins of MSMEG_5634 and analyze the conserved domains among them. An evolutionary tree was constructed as shown in Figure 2A. MSMEG_5634 and its homologous proteins are highly conserved among mycobacteria and belong to the SRPBCC (START/RHOs_alpha_C/PITPs/Bet v1/CoxG/CalC) superfamily, containing a START domain that functions as a lipid-binding domain involved in eukaryotic signal transduction as well as a cyclase/aromatase domain for the biosynthesis of polyketide antibiotics in Actinomycetes.

MSMEG_5634 was overexpressed in *E. coli*, purified as described in the Materials and Methods, and then subjected to crystallization (Supplementary Figure S4A). The structure of MSMEG_5634 was solved at 1.9 Å resolution (Figure 2B; Supplementary Table S1). MSMEG_5634 has a helix-grip structure that contains seven-stranded antiparallel β-sheets (named β1 to β7) and four α-helices (α1 to α4). α1 and α2 are two short consecutive helices located between β1 and β2, and α3 is also a short α-helix which is followed by the long C-terminal helix α4 (Figure 2B). The seven-stranded antiparallel β-sheets and the long C-terminal α-helix α4 together fold into a barrel, with a deep hydrophobic pocket wrapped in the middle of the barrel. The first two short helices α1 and α2 fold into a V-shaped structure that seals the bottom of the pocket (Figure 2B). The overall structure of MSMEG_5634 is similar to that of polyketide cyclase/aromatase TcmN ARO/CYC (PDB: 3TVQ; Ames et al., 2008). However, when the structure of MSMEG_5634 is superimposed with that of TcmN ARO/CYC, an average rmsd of 5.750 Å was shown for the backbone atoms, indicating distinct structure variations (Figure 2C, left panel). The differences were mainly shown in the following three aspects. First, regarding to the structural elements, MSMEG_5634 has a long flexible loop between β2 and β3, while the corresponding loop in TcmN ARO/CYC is shorter (Figure 2C, middle panel). MSMEG_5634 has a small C-terminal α-helix (α3) at the opening of the hydrophobic pocket, but TcmN ARO/CYC does not (Figure 2C, right panel). Second, regarding the catalytic active center of TcmN ARO/CYC, the cyclization or aromatization of polyketides is mediated by Tyr-35, Arg-69, and Arg-82 (Ames et al., 2008). According to the protein sequence and structure, the corresponding residues of MSMEG_5634 are Tyr-27, Arg-63, -65, and -84, but the side chains of Arg-63, -65, and -84 all point to the outside of the pocket (Figure 2D). Third, the ligand-binding pocket of MSMEG_5634 is more hydrophobic than the pocket of TcmN ARO/CYC (Supplementary Figure S4B). A clear linear electron density was observed inserted into the pocket of MSMEG_5634, which might be a chain of a fatty acid based on its shape (Figure 2E), indicating that MSMEG_5634 may bind fatty acid chain-containing substrate.

### MSMEG_5634 Interacts With AcpM and the FAS-II Complex

**Figure 3:**
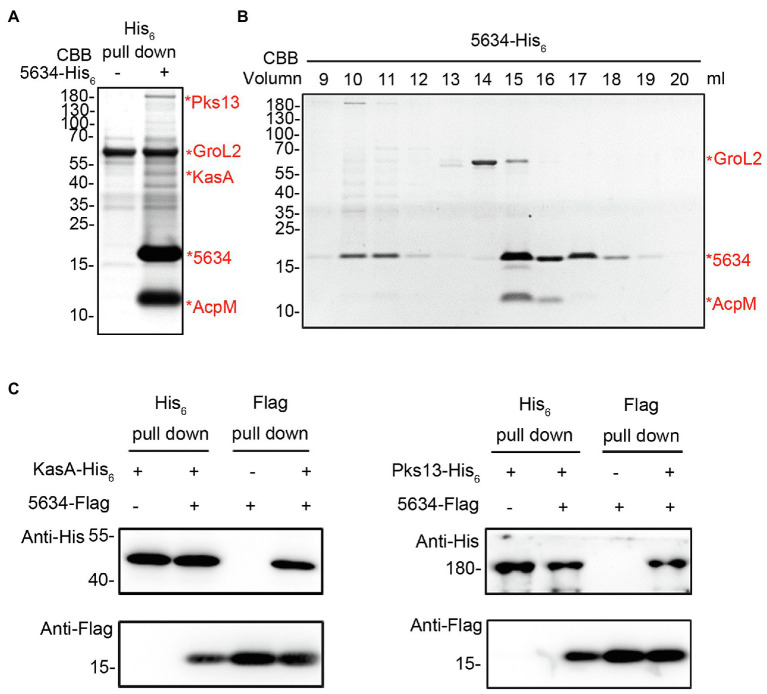
Interactions between MSMEG_5634, AcpM, and FAS-II components. **(A)** Identification of proteins interacting with MSMEG_5634 in *Mycobacterium smegmatis*. MSMEG_5634-His_6_ was expressed in *M. smegmatis* and purified by Ni^2+^-NTA affinity chromatography. The eluted proteins were collected and analyzed by Tricine-SDS-PAGE. The indicated protein bands were excised, digested with trypsin, and then analyzed by MALDI-TOF. **(B)** The total protein sample copurified with MSMEG_5634-His_6_ expressed in *M. smegmatis* was subjected to size exclusion chromatography on a Superdex 200 Increase 10/300 Gl column. The resulting fractions were collected every 1 ml and then analyzed by Tricine-SDS-PAGE. **(C)** The interactions between MSMEG_5634 and KasA and Pks13 were detected by pull-down assays. Flag-tagged MSMEG_5634 was coexpressed with His_6_-tagged KasA (left panel) or His_6_-tagged Pks13 (right panel) in *E. coli*. Flag-tagged MSMEG_5634 was purified by Flag affinity chromatography, and His_6_-tagged KasA/Pks13 was purified by Ni^2+^-NTA affinity chromatography, respectively. The eluted proteins were analyzed by SDS-PAGE and Western blot.

To further understand the biological roles of MSMEG_5634, pull-down assays were performed to detect the proteins interacting with MSMEG_5634 in *M. smegmatis*. His_6_-tagged MSMEG_5634 was expressed in *M. smegmatis* and then purified by Ni^2+^-NTA affinity chromatography. MSMEG_5634 was apparently copurified with a protein with a molecular weight of approximately 10 kDa, which was identified as a meromycolate extension acyl carrier protein (AcpM) by mass spectrometry (Figure 3A; Supplementary Table S2). Among other proteins copurified with MSMEG_5634, a protein of approximately 45 kDa was identified as 3-oxoacyl-[acyl carrier protein] synthase 1 (KasA), and a protein of approximately 180 kDa was identified as polyketide synthase 13 (Pks13), both of which belong to the FAS-II system (Figure 3A; Supplementary Table S2). Meanwhile, a protein of approximately 60 kDa was identified GroL2 (also known as CH60 2) protein, which was pulled-down as a contaminant protein during the Ni^2+^-NTA affinity chromatography (Figure 3A; Supplementary Table S2). In further studies, the total protein sample copurified with His_6_-tagged MSMEG_5634 expressed in *M. smegmatis* was subjected to size exclusion chromatography and then analyzed by Tricine-SDS-PAGE. MSMEG_5634 was present in three major fractions (Figure 3B; Supplementary Figure S5): the MSMEG_5634 protein alone, the complex of MSMEG_5634 and AcpM, and MSMEG_5634 combined with a number of proteins, among which FAS-II enzymes, such as Pks13, KasA, and InhA, were detected when the protein components were subjected to mass spectrometry analysis (Supplementary Table S3). When Flag-tagged MSMEG_5634 was coexpressed with His_6_-tagged Pks13 or His_6_-tagged KasA in *E. coli*, it was found that MSMEG_5634 could interact with either Pks13 or KasA directly by pull-down assays (Figure 3C; Supplementary Figure S6). These results suggest that MSMEG_5634 can interact with FAS-II components and form an MSMEG_5634/AcpM complex that is free from FAS-II.

### MSMEG_5634 Interacts With Acyl-AcpM Containing a Long Fatty Acid Chain

**Figure 4:**
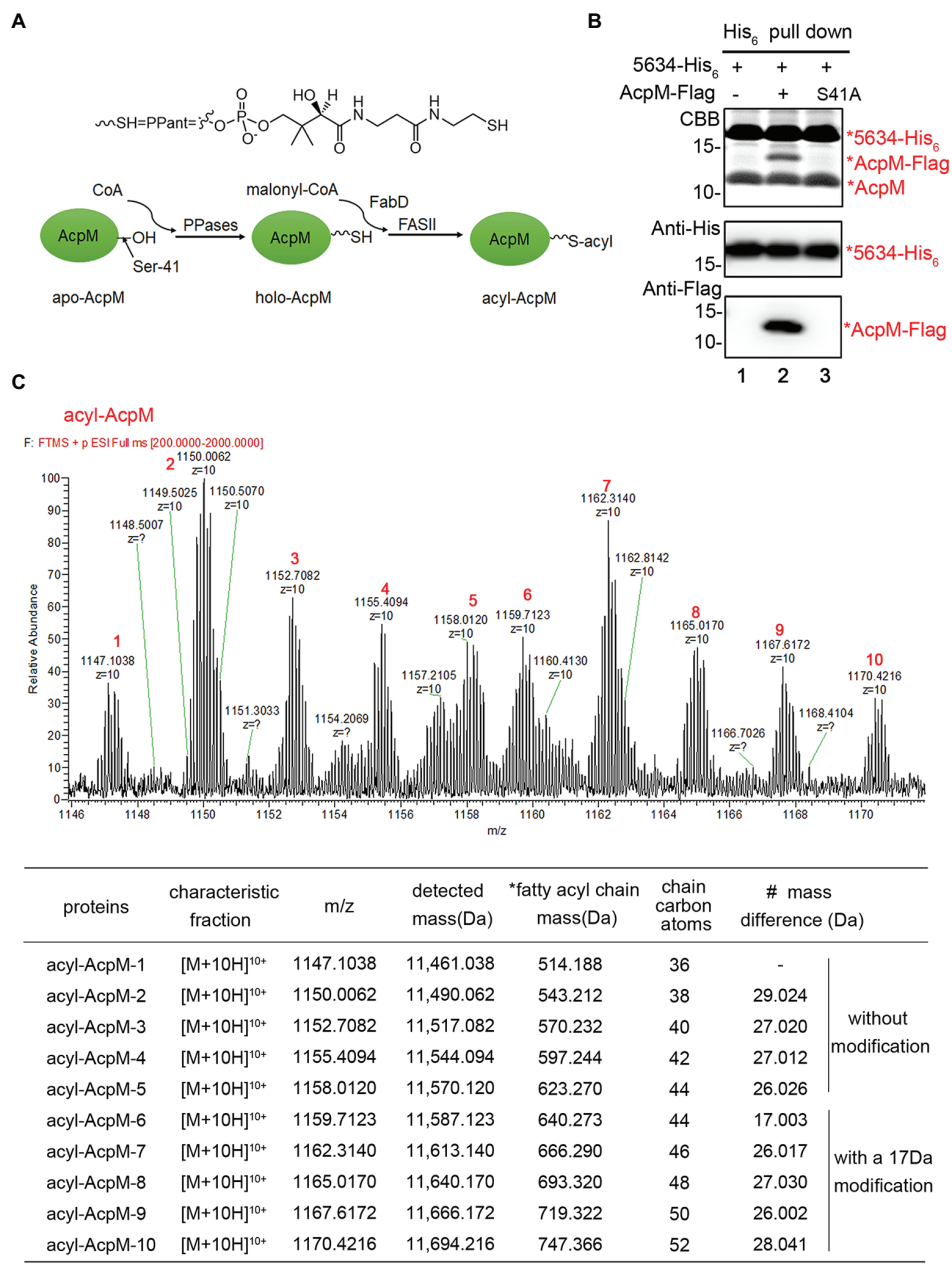
MSMEG_5634 interacts with acyl-AcpM containing a long fatty acid chain. **(A)** Schematic diagram showing the formation process of acyl-AcpM. **(B)** MSMEG_5634-His_6_ was coexpressed with AcpM-Flag (Lane 2) or AcpM (S41A)-Flag (Lane 3) in *Mycobacterium smegmatis*. MSMEG_5634-His_6_ was purified by Ni^2+^-NTA affinity chromatography. The eluted proteins were collected and analyzed by Tricine-SDS-PAGE and Western blot. **(C)** Determination of the mass of acyl-AcpM interacting with MSMEG_5634-His_6_ by UPLC-HR-MS. The detailed data are listed under the mass spectrometry. *Fatty acyl chain mass, the mass of the fatty acyl chain in acyl-AcpM which is calculated by subtracting the theoretical molecular weight of holo-AcpM 10,946.85 from the molecular weight of acyl-AcpM. # Mass difference, the increase in the mass of fatty acyl chain compared with the value in the above line.

AcpM plays a central role in transporting fatty acid intermediates in the FAS-II system. There are three types of AcpM, apo-AcpM, holo-AcpM, and acyl-AcpM, during the fatty acid elongation in mycobacterium (Figure 4A). The conserved serine residue (Ser-41) of apo-AcpM is posttranslationally modified by a phosphopantetheine arm to form holo-AcpM, and then, the malonate moiety from malonyl-CoA is transferred to holo-AcpM by FabD to form malonyl-AcpM (Kremer et al., 2001). Acyl-AcpM with a long fatty acyl chain is produced through the condensation of malonyl-AcpM with acyl-CoA and the subsequent elongation by FAS-II (Schaeffer et al., 2001; Wong et al., 2002; Figure 4A). We constructed an AcpM mutant with serine 41 substituted by alanine, termed AcpM (S41A). When AcpM (S41A) was expressed in *M. smegmatis*, it was found that MSMEG_5634 was not able to interact with AcpM (S41A) (Figure 4B), indicating that the complex formation of MSMEG_5634 and AcpM depends on the further modification of AcpM.

The molecular weights of MSMEG_5634-His_6_ and acyl-AcpM in the MSMEG_5634-His_6_/acyl-AcpM complex were determined by mass spectrometry. The average accurate mass of MSMEG_5634-His_6_ in the complex was determined to be 17,090.834 Da (Supplementary Figure S7), which is consistent with its theoretical molecular weight of 17,095.599 Da. The acyl-AcpM molecules in the complex were detected with masses between 11,461.038 and 11,694.216 Da. It is interesting to note that the mass of acyl-AcpM increases by a multiple of approximately 28, which is matched with the common rule of fatty acid elongation of its molecular weight plus 28 per cycle with the addition of a two-carbon unit (Figure 4C). The mass of the fatty acyl chain in acyl-AcpM can be calculated by subtracting the molecular weight of holo-AcpM, which is theoretically 10,946.85, from the molecular weight of acyl-AcpM. According to mass data, the length of the fatty acyl chain in acyl-AcpM was estimated to be between C36 and C52, and a modification may occur on the fatty acyl chains at the C44-52 length, leading to an addition of approximately 17 Da in molecular mass. These results indicate that MSMEG_5634 prefers to form a complex with acyl-AcpM containing a fatty acid at the C36-52 length, which is the intermediate product in fatty acid elongation by FAS-II.

Rv0910 is the homologous protein of MSMEG_5634 in *M. tuberculosis* with an identity of 67.35% (Supplementary Figure S8A) and lies at a similar gene locus as MSMEG_5634 (Supplementary Figure S9A). Ectopic expression of Rv0910 did not inhibit the growth of *M. smegmatis* (Supplementary Figures S9B,C). It was found that Rv0910 could form a complex with AcpM; however, MSMEG_3418, the homologous protein of MSMEG_5634 in *M. smegmatis* with an identity of 36.73%, hardly interacted with AcpM (Supplementary Figure S8). These data suggest that Rv0910 and MSMEG_5634 might have similar biological functions.

### Cellular Location of MSMEG_5634 in *Mycobacterium smegmatis*

**Figure 5:**
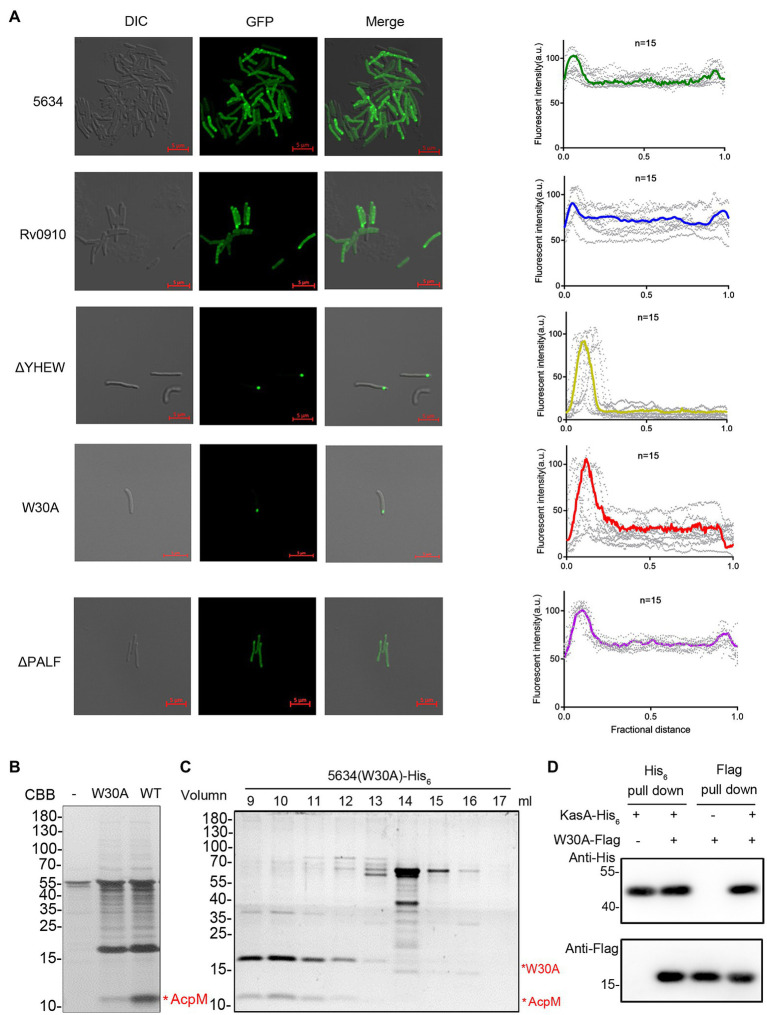
Cellular locations of MSMEG_5634, its mutants, and its homologous protein. **(A)** MSMEG_5634, its homologous protein Rv0910, and MSMEG_5634 ΔPALF (113–116 residues of the α3 helix), ΔYHEW (27–30 residues of the α2 helix), and W30A mutants were expressed with GFP fused at the C-terminus in *Mycobacterium smegmatis*. The cellular locations of these GFP fusion proteins were detected by confocal microscopy. The fluorescence quantification over the long axis of cells was performed and analyzed by ImageJ profile. **(B)** MSMEG_5634-His_6_ and MSMEG_5634 (W30A)-His_6_ were expressed in *M. smegmatis* and purified by Ni^2+^-NTA affinity chromatography, respectively. The eluted proteins were collected and analyzed by Tricine-SDS-PAGE. **(C)** The total protein sample copurified with MSMEG_5634 (W30A)-His_6_ expressed in *M. smegmatis* was subjected to size exclusion chromatography. The resulting fractions were collected every 1 ml and then analyzed by Tricine-SDS-PAGE. **(D)** The interaction between MSMEG_5634 (W30A) and KasA was detected by pull-down assays. Flag-tagged MSMEG_5634 (W30A) was coexpressed with His_6_-tagged KasA in *E. coli*. Flag-tagged MSMEG_5634 (W30A) was purified by Flag affinity chromatography, and His_6_-tagged KasA was purified by Ni^2+^-NTA affinity chromatography, respectively. The eluted proteins were analyzed by SDS-PAGE and Western blot.

In mycobacteria, the FAS-II complex, which plays an important role in mycolic acid synthesis, is specifically located at the polar site to produce mycolic acids for bacterial growth (Carel et al., 2014). MSMEG_5634 protein fused with GFP at the C-terminus (MSMEG_5634-GFP) was expressed in *M. smegmatis*, and then, the cellular location of MSMEG_5634-GFP was detected by confocal microscopy. Upon quantifying the distribution of fluorescence, we observed that MSMEG_5634-GFP was diffusely distributed in the cytoplasm with relative accumulation at the poles (Figure 5A). Rv0910-GFP expressed in *M. smegmatis* displayed a cellular location similar to MSMEG_5634-GFP (Figure 5A). When residues 27–30 of YHEW in helix α2 were deleted, the MSMEG_5634 mutant, termed ΔYHEW, was highly accumulated at the pole, but the deletion of residues 113–116 of PALF in helix α3, termed ΔPALF, had no impact on MSMEG_5634 cellular location (Figure 5A). When hydrophobic Trp-30 in helix α2 was mutated to alanine, the MSMEG_5634 (W30A) mutant obviously accumulated at the polar region (Figure 5A). Much less AcpM was copurified with His_6_-tagged MSMEG_5634 (W30A) expressed in *M. smegmatis* by Ni^2+^-NTA affinity chromatography (Figure 5B). When the total protein sample copurified with His_6_-tagged MSMEG_5634 (W30A) was subjected to size exclusion chromatography and then analyzed by Tricine-SDS-PAGE, it was found that MSMEG_5634 (W30A) was mainly present in the high molecular weight multiprotein fractions containing FAS-II interconnected enzymes and acyl-AcpM (Figure 5C). Compared with the wild-type MSMEG_5634 (Figure 3B), MSMEG_5634 W30A retains the ability to associate to FAS-II complex but loses the ability to interact with acyl-AcpM to form a complex free from FAS-II, suggesting that W30 residue in MSMEG_5634 plays an essential role in its interaction with acyl-AcpM. When Flag-tagged MSMEG_5634 (W30A) was coexpressed with His_6_-tagged KasA in *E. coli*, it was found that MSMEG_5634 (W30A) could interact with KasA by pull-down assay (Figure 5D). These data suggest that MSMEG_5634 (W30A) can interact with the FAS-II component but cannot form a complex with acyl-AcpM to transport acyl-AcpM from FAS-II to cytoplasm.

### Deletion of the *MSMEG_5634* Gene Decreases FAS-II Inhibitor Susceptibility

**Figure 6:**
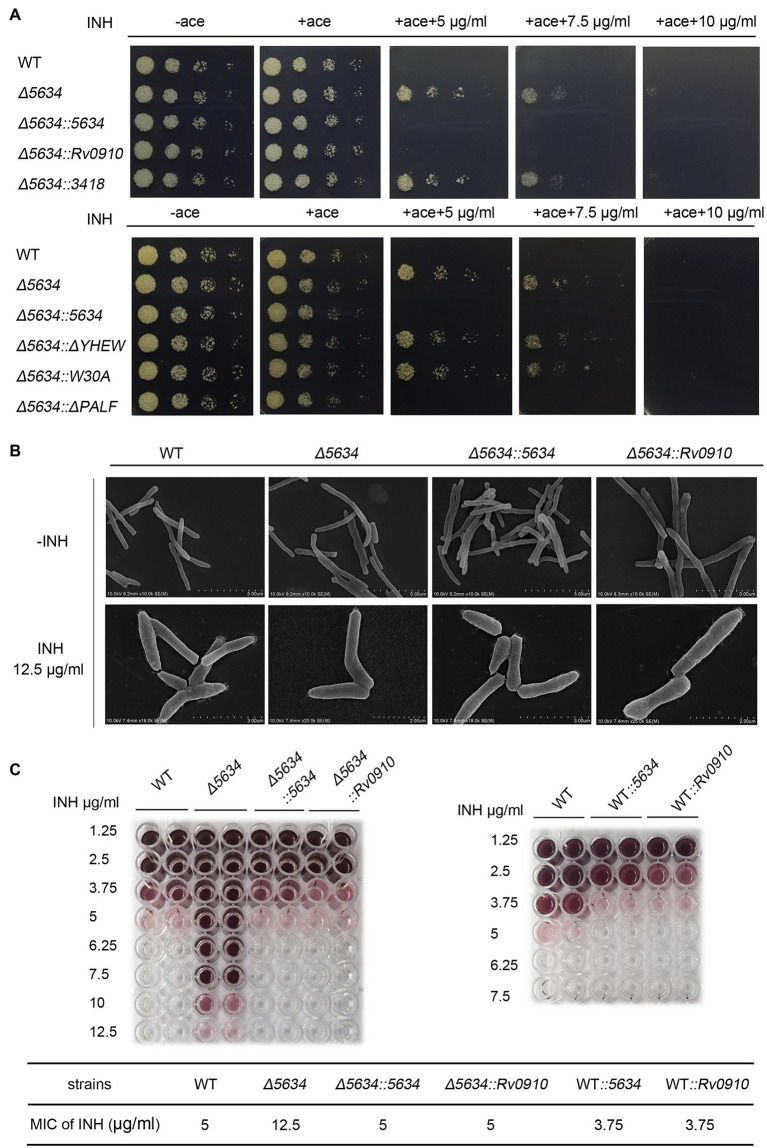
The impacts of MSMEG_5634 on the isoniazid sensitivity in *Mycobacterium smegmatis*. **(A)** The indicated *M. smegmatis* cells were serially diluted and grown on 7H10 solid medium with different concentrations of isoniazid (INH) in the presence or absence of acetamide (0.2%). The cells untreated with isoniazid were cultured for 48 h, and the cells treated with INH were cultured for 72 h. **(B)** Morphology detection by scanning electron microscopy. The indicated *M. smegmatis* strains were treated with 12.5 μg/ml INH for 12 h in the presence of acetamide (0.2%). **(C)** The minimum inhibitory concentrations of INH to different strains. The indicated strains (1 × 10^5^ cells/well) were cultured in 7H9 medium for 24 h at 37°C under INH treatment at different concentrations in the presence of acetamide (0.2%) with TTC (0.02%) as the indicator. WT, the wild-type cells containing empty pACE vector; *Δ5634*, the *MSMEG_5634*-deleted cells containing empty pACE FIGURE 6vector; *Δ5634::5634*, *Δ5634::Rv0910* and *Δ5634::3418* are the *MSMEG_5634* gene-deleted cells with the ectopic expression of MSMEG_5634, Rv0910, MSMEG_3418, respectively, under the control of the inducible acetamidase promoter in the pACE vector; *Δ5634::ΔYHEW* and *Δ5634::W30A* are the *MSMEG_5634*-deleted cells with the ectopic expression of the indicated MSMEG_5634 mutant in the presence of acetamide (ace, 0.2%); and WT*::5634* and WT*::Rv0910* are the wild-type cells with the ectopic expression of MSMEG_5634, and Rv0910, respectively, under the control of the inducible acetamidase promoter in the pACE vector.

The *M. smegmatis* strain with *MSMEG_5634* gene deletion (∆5634) was constructed by homologous recombination (Supplementary Figure S10A). The deletion of *MSMEG_5634* gene in genomic DNA was identified by PCR and DNA sequencing (Supplementary Figure S10B). *MSMEG_5634* gene deletion had no significant impact on *M. smegmatis* growth under normal conditions (Supplementary Figure S10C). Since MSMEG_5634 can interact with FAS-II components and form a complex with acyl-AcpM, it may play a role in mycolic acid synthesis, the vital process targeted by anti-tuberculosis drugs such as the FAS-II inhibitor isoniazid (Miesel et al., 1998; Rafi et al., 2006). Therefore, the effects of MSMEG_5634 gene deletion on isoniazid sensitivity were detected. It was found that the ∆5634 strain became tolerant to isoniazid. The isoniazid sensitivity of the ∆5634 strain was restored by the ectopic expression of MSMEG_5634 but not by the ectopic expression of MSMEG_5634 (ΔYHEW) and MSMEG_5634 (W30A) mutants, in which the α2 helix was disrupted by deletion or point mutation, respectively (Figure 6A). The discrepancy of these complemented strains was not due to the difference in expression levels between wild-type MSMEG_5634 and its mutants as detected by Western blot (data not shown). As mentioned above, compared with wild-type MSMEG_5634, MSMEG_5634 (W30A) is unable to interact with acyl-AcpM to form a complex free from FAS-II. Therefore, we propose that the transportation of acyl-AcpM away from FAS-II by forming an MSMEG_5634/acyl-AcpM complex may contribute to susceptibility to isoniazid.

When detected by scanning electron microscopy, the wild-type strain and the ∆5634 strain did not show different morphologies under normal conditions. It is known that the inhibition of mycolic acid synthesis by isoniazid results in morphological changes in the mycobacteria. Under isoniazid (12.5 μg/ml) treatment, the surface of wild-type *M. smegmatis* was disturbed, while the surface of the ∆5634 strain remained smooth. When the ∆5634 strain was complemented with MSMEG_5634 expression, under isoniazid treatment, its cell walls became as crude as those of the wild-type strain (Figure 6B).

**Figure 7:**
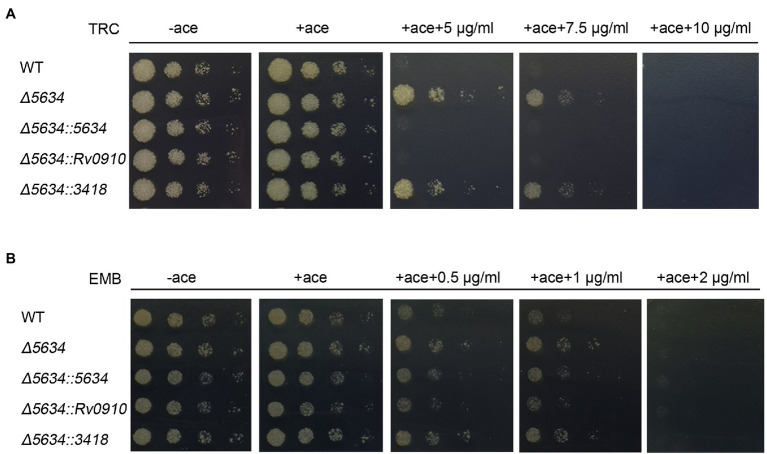
*MSMEG_5634* deletion decreases sensitivity to triclosan (TRC) but not to ethambutol (EMB) in *Mycobacterium smegmatis*. The indicated *M. smegmatis* cells were serially diluted and grown on 7H10 medium with different concentrations of TRC **(A)** and EMB **(B)**. The cells untreated with drug were cultured for 48 h, and the strains treated with drug were cultured for 72 h. WT, wild-type cells containing empty pACE vector; *Δ5634*, the *MSMEG_5634*-deleted cells containing empty pACE vector; and *Δ5634::5634*, *Δ5634::Rv0910* and *Δ5634::3418* are the *MSMEG_5634*-deleted cells with the ectopic expression of MSMEG_5634, Rv0910, MSMEG_3418, respectively, under the control of the inducible acetamidase promoter in pACE vector in the presence of acetamide (ace, 0.2%).

The impacts of *MSMEG_5634* deletion on susceptibility to other antibiotics were further detected. Deletion of *MSMEG_5634* also induced tolerance to triclosan (TRC), another anti-tuberculosis drug that targets InhA to block FAS-II and inhibits mycolic acid synthesis (Slayden et al., 2000). However, MSMEG_5634 deletion had no impact on the sensitivity to ethambutol, which inhibits the synthesis of the arabinose polymer (Schubert et al., 2017; Figure 7). When the ∆5634 strain was complemented with the expression of *MSMEG_5634* homologous gene, it was found that the ectopic expression of *Rv0910*, but not *MSMEG_3418*, could resume sensitivity to isoniazid (Figure 6A) and triclosan (Figure 7A). Under isoniazid treatment, the ∆5634 strain complemented with Rv0910 expression had the same morphological change as the wild-type *M. smegmatis* (Figure 6B). The minimum inhibitory concentration (MIC) of isoniazid was increased from 5 μg/ml in the wild-type strain to 12.5 μg/ml in the ∆5634 strain and was restored to 5 μg/ml when the ∆5634 strain was complemented with MSMEG_5634 or Rv0910 expression. Conversely, in the wild-type *M. smegmatis* strain, the MIC of isoniazid was decreased from 5 to 3.75 μg/ml by the overexpression of either MSMEG_5634 or Rv0910 (Figure 6C). The same phenomena were observed when the MIC of triclosan was tested in these strains (Supplementary Figure S11). These data suggest that MSMEG_5634 and Rv0910, its homolog in *M. tuberculosis*, contribute to the intrinsic susceptibility of anti-tuberculosis drugs targeting FAS-II.

## Discussion

### MSMEG_5634 Is a Novel Acyl-AcpM-Binding Protein

MSMEG_5634 is a hypothetical protein in *M. smegmatis* that has been proposed as a toxic member that forms a toxin-antitoxin system with MSMEG_5635 to regulate bacterial growth (Ramage et al., 2009). In this paper, we found that the ectopic expression of MSMEG_5634 did not have inhibitory effects on the growth of *M. smegmatis* and that MSMEG_5634 could not interact with MSMEG_5635 to form a toxin-antitoxin complex. Instead, MSMEG_5634 was able to directly interact with acyl-AcpM. MSMEG_5634 could not bind with AcpM (S41A), the mutant that cannot be acylated, indicating that the fatty acyl chain in acyl-AcpM is required for the interaction. Notably, MSMEG_5634 prefers to form a complex with acyl-AcpM protein containing a fatty acyl chain at the length of C36-52, which is the intermediate product of meromycolate chain synthesis by FAS-II. In the acyl-AcpM proteins interacting with MSMEG_5634, a modification with a molecular mass of approximately 17 Da was detected on the fatty acyl chains at the C44-52 length, which is guessed to be hydroxylation but needs further clarification. MSMEG_5634 exists in three forms, as detected by gel filtration chromatography: free MSMEG_5634 protein, MSMEG_5634/acyl-AcpM complex, or in a multiprotein fraction with FAS-II components. With pull-down assays, it was found that MSMEG_5634 could directly interact with the FAS II components KasA and Pks13. It has been reported that FAS-II has a polar location (Carel et al., 2014). The cellular location of MSMEG_5634 was found at the pole as well as in the cytoplasm, which is consistent with our results by gel filtration chromatography. The interaction between acyl-AcpM and the individual FAS-II component is transient and reversible during fatty acid elongation, while the interaction between acyl-AcpM and MSMEG_5634 is somehow much stable. We propose that MSMEG_5634 interacts with the FAS-II complex and transports acyl-AcpM away from FAS-II by forming an MSMEG-5634/acyl-AcpM complex. However, the ectopic expression of MSMEG_5634 does not impact *M. smegmatis* cell growth under normal culture conditions, which may be due to the abundance of acyl-AcpM.

### Structure of the MSMEG_5634 Protein

MSMEG_5634 is a member of the SRPBCC superfamily. In this paper, it was determined that MSMEG_5634 has a single helix-grip-fold START domain containing a deep hydrophobic pocket formed by seven antiparallel β-sheets (β1–7) and a long α-helix (α4). The START domain, initially identified as a lipid-binding domain involved in eukaryotic signal transduction, is also present in bacterial proteins, including cyclases/aromatases involved in the biosynthesis of polyketide antibiotics in Actinomycetes. The overall structure of MSMEG_5634 is similar to that of the TcmN polyketide cyclase/aromatase domain but with differences in the conformation of proposed active site residues. In the refined structure of the MSMEG_5634 protein expressed and purified from *E. coli*, there was a linear electron density, which might be a chain of fatty acid inserted into the pocket of MSMEG_5634. MSMEG_5634 does not interact with acyl-ACP *in E. coli* but prefers to form a complex with acyl-AcpM containing the long fatty acyl chain (C36–52) in *M. smegmatis*. To form the complex, the fatty acyl chain of acyl-AcpM may be translocated from the hydrophobic core of AcpM and insert into the hydrophobic binding pocket of MSMEG_5634. The interaction between MSMEG_5634 and acyl-AcpM was disrupted by the substitution mutation of W30A in helix α2, which formed a helix–loop–helix motif together with the α1 helix at the bottom of the pocket. The MSMEG_5634 (W30A) and ΔYHEW (helix α2 deletion) mutants display polar accumulation in cells, suggesting that the interaction with acyl-AcpM may induce the conformational change of MSMEG_5634 protein, which is required to release the MSMEG_5634/acyl-AcpM complex from FAS-II. Therefore, the START domain of MSMEG_5634 performs a special biological function in the transportation of acyl-AcpM in *M. smegmatis*.

### Biological Function of MSMEG_5634 and Its Homologous Proteins

MSMEG_5634 and its homologous proteins are highly conserved in mycobacterium. Rv0910, the MSMEG_5634 homologous protein in *M. tuberculosis*, was able to interact with AcpM to form a complex and displays a polar and cytoplasmic location in cells. In this paper, the *M. smegmatis* strain with *MSMEG_5634* deletion (∆5634) was constructed. Deletion of the *MSMEG_5634* gene did not impact *M. smegmatis* growth under normal culture conditions. However, compared to the wild-type strain, the ∆5634 strain was less sensitive to isoniazid or triclosan treatment, both of which are well-known drugs targeting FAS-II to block mycolic acid synthesis. Meanwhile, MSMEG_5634 deletion had no effects on sensitivity to ethambutol (EMB). The isoniazid and triclosan sensitivities were restored when the ∆5634 strain was complemented with the expression of either MSMEG_5634 or Rv0910. The ectopic expression of MSMEG_5634 (W30A), which loses the ability to transport acyl-AcpM away from FAS-II, could not restore the sensitivity to isoniazid and triclosan in the ∆5634 strain. Based on these results, we propose that under treatment with a FAS-II inhibitor, MSMEG_ 5634 and Rv0910 further inhibit mycotic acid synthesis by releasing acyl-AcpM from FAS-II, thereby improving drug sensitivity.

**Figure 8:**
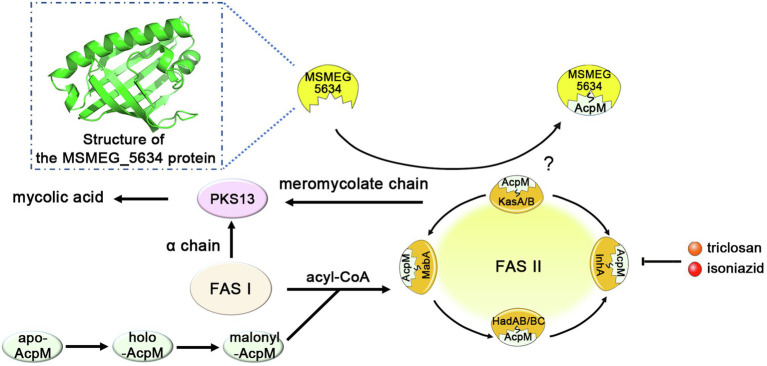
A model depicting the structure and function of MSMEG_5634. MSMEG_5634 was determined to be a helix-grip structure protein with a deep hydrophobic pocket, preferring to form a complex with acyl-AcpM containing a fatty acyl chain at the C36-52 length, which is the medium product of FAS-II. MSMEG_5634 can interact with FAS-II components and transport acyl-AcpM away from FAS-II by forming the MSMEG_5634/acyl-AcpM complex, which is free from FAS-II. MSMEG_5634 contributes to the susceptibility of the drugs targeting FAS-II. Deletion of the *MSMEG_5634* gene in *Mycobacterium smegmatis* leads to the tolerance to isoniazid and triclosan, two inhibitors of the FAS-II system. The question marker indicates the impact of MSMEG_5634 on FAS-II activity, especially under stress conditions, remains to be further studied.

In conclusion, in this paper, we determined that MSMEG_5634 is a START domain protein with a helix-grip-fold structure and identified it as a novel acyl-AcpM binding protein, preferring to form a complex with acyl-AcpM containing a fatty acyl chain at the C36-52 length, which is the medium product of FAS-II. MSMEG_5634 can transport acyl-AcpM away from FAS-II by forming the MSMEG_5634/acyl-AcpM complex and contributes to intrinsic sensitivity to FAS-II inhibitors. However, the impacts of MSMEG_5634 on FAS-II activity and *de novo* mycolic acid biosynthesis have not been tested in this study (Figure 8). Neither deletion nor overexpression of MSMEG_5634 has obvious impact on the growth of *M. smegmatis* under normal culture conditions (Figure 1; Supplementary Figure S9), suggesting that MSMEG_5634 may has no significant effects on FAS-II activity under normal culture conditions. The role of MSMEG_5634 in FAS-II activity regulation, especially under stress conditions, remains to be further studied. MSMEG_5634 and its homologous proteins form a conserved family in mycobacteria. It has been reported that the expression of Rv0910, the MSMEG_5634 homologues in *M. tuberculosis*, was decreased under either hypoxia or nutrient starvation (Betts et al., 2002; Del Portillo et al., 2018). Thus far, it cannot be ruled out that in addition to impacting the sensitivity of FAS-II inhibitors, MSMEG_5634 and its homologs, such as Rv0910 in *M. tuberculosis*, may have other biological functions, which also need to be further explored.

## Data Availability Statement

The datasets presented in this study can be found in online repositories. The names of the repository/repositories and accession number(s) can be found in the article/Supplementary Material.

## Author Contributions

JZ designed the experiments and analyzed the data. ML performed most experiments. QH contributed to the construction of the MSMEG_5634 gene deletion strain and drug sensitivity analysis. WZ, YC, and ZW contributed to protein crystallization and structure analysis. ZZ contributed to mass spectrometry analysis. XZ provided technical guidance. ML and JZ wrote the manuscript. All authors contributed to the article and approved the submitted version.

## Funding

This work was supported by grants from the National Natural Science Foundation of China (NSFC; nos. 81972604 and 32171138) and a grant from the Natural Science Foundation of Beijing Municipality (no. 7192102) to JZ, and by grants from NSFC (nos. 31870725 and 32071204) to ZW.

## Conflict of Interest

The authors declare that the research was conducted in the absence of any commercial or financial relationships that could be construed as a potential conflict of interest.

## Publisher’s Note

All claims expressed in this article are solely those of the authors and do not necessarily represent those of their affiliated organizations, or those of the publisher, the editors and the reviewers. Any product that may be evaluated in this article, or claim that may be made by its manufacturer, is not guaranteed or endorsed by the publisher.

## Footnotes

^1^ www.matrixscience.com
