# Supplementary material for: A Novel Acyl-AcpM-Binding Protein Confers Intrinsic Sensitivity to Fatty Acid Synthase Type II Inhibitors in Mycobacterium smegmatis
Source: Front Microbiol. 2022 Apr 4;13:846722. doi: 10.3389/fmicb.2022.846722 (PMC9014085; doi:10.3389/fmicb.2022.846722)
Supplement: Supplementary file 1 [file Data_Sheet_1.docx]

Supplementary Material

# Materials and Methods

**Construction of the *MSMEG_5634*-deleted *Mycobacterium smegmatis* strain**

The *Mycobacterium smegmatis strain* with *MSMEG_5634* gene deletion was constructed by homologous recombination using the pNIL/pGOAL system. A 1264-bp upstream region of the *MSMEG_5634* gene was chosen to be the right arm, and a 1236-bp downstream region of the *MSMEG_5634* gene was chosen to be the left arm. The positions of the left and right arms are shown in Fig. S10A. The left and right arms were inserted into the p1NIL vector to create the p1NIL 5634 L/R vector. The p1NIL 5634 L/R and pGOAL19 vectors were digested with Pac I. The marker cassette (hyg, lacZ, sacB) from pGOAL19 was linked with the Pac I-digested p1NIL 5634 L/R vector to create the p1NIL/pGOAL19 5634 L/R vector, which was electroporated into *M. smegmatis* cells. Strains with plasmid marker genes (kan, hyg, lacZ) were selected, in which a single recombination event had been done, termed single crossover strains (SCOs). SCOs were streaked onto 7H10/glycerol/OADC without antibiotics to allow the second recombination event to occur, creating double crossover strains (DCOs), which can be selected in the presence of 2% sucrose and X-gal by forming white and sucrose^R^ colonies. Finally, the colonies were tested for sensitivity to kanamycin, and the kan^S^ colonies were further screened by colony PCR to identify the MSMEG_5634 deletion strain. The MSMEG_5634 gene (444 bp) was completely deleted from *M. smegmatis* genomic DNA. The deletion of the *MSMEG_5634* gene in genomic DNA was confirmed by DNA sequencing.

The primers used for MSMEG_5634 gene deletion were as follows:

Δ5634-up-Kpn I-F, 5´-GGGGTACCCAAGCTGCACCCGCGGGA-3´;

Δ5634-up-R, 5´-CAGTAACACACGCACAGATCAGCCTGTAACCCGCT-3´;

Δ5634-down-F, 5´-GGTTACAGGCTGATCTGTGCTGGTGTTACTGCTGTG-3´;

Δ5634-down-Hind III-R, 5´-CCCAAGCTTCTCGACTACTTCGCGTCCC-3´.

The primers used to identify the MSMEG_5634-deleted strain were as follows:

Δ5634-F, 5´- ATCCACAAAGACGACCCTCA-3´;

Δ5634-R, 5´- TACGGCAAGGTGCTCAAGCG -3´.

***RNA isolation and reverse transcriptase PCR***

Bacteria were pelleted and lysed by bead beating with 425-600 μm glass beads (Sigma, America) in 500 μl Trizol at 5000 m/s speed for 90 s, and total RNA was isolated via chloroform extraction and sodium acetate precipitation as previously described ([Voskuil et al., 2003](#_ENREF_3)). RNA (1.0 μg) treated with DNase I were prepared for with reverse transcription. The primers used for reverse transcriptase PCR analysis of MSMEG_5635/5634 operon were list below.

Primers used to amplify *MSMEG_5634* and *MSMEG_5635*:

RT-PCR-5634-F, 5' -TATGCCTCCGACCTGTCCC-3';

RT-PCR-5634-R, 5' -GGCCGTCACCGTTGAGTGT-3';

RT-PCR-5635-F, 5' -ATGGGATTCCTGGACAAGG-3';

RT-PCR-5635-R, 5' -CTGCTGTGGTTCTTGAGGGT-3'

Primers used to amplify the region between *MSMEG_5634* and *MSMEG_5635*:

RT-PCR-5635-5634-F, 5' -ATGGGATTCCTGGACAAGG-3'

RT-PCR-5635-5634-R, 5' -GGCCGTCACCGTTGAGTGT-3'

Primers used to amplify the region between *MSMEG_5635* and *MSMEG_5636:*

RT-PCR-5636-5635-F, 5' -CAGCCCATCCACGTCACCA-3'

RT-PCR-5636-5635-R, 5' -CTGCTGTGGTTCTTGAGGGT-3'

***Quantitative confocal microscopy imaging and statistics analysis***

Confocal images were obtained on a Zeiss LSM880-UV microscope. GFP fluorescence was excited with the 488 nm argon laser lines; Emitted light was collected through 505- to 530-nm (GFP) band-pass filters. Bright-field images were collected with a transmitted light detector. Laser intensity, photomultiplier gain, and offset were standardized. The statistics analysis referred to method described before ([Meniche et al., 2014](#_ENREF_1)). The cells without septum were chosen to be analyzed. The fluorescence intensity per pixel along the cell lengths (or segments) were analyzed by Image J profile. Because the cell lengths were variable, the segment lengths were normalized to yield a fractional distance. The fractional distance and fluorescence intensity were combined into one dataset and sorted on fractional distance. The average of different segments was calculated from the dataset by using a window size equal to the number of segments.

# Supplementary Figures and Tables

## Supplementary Figures


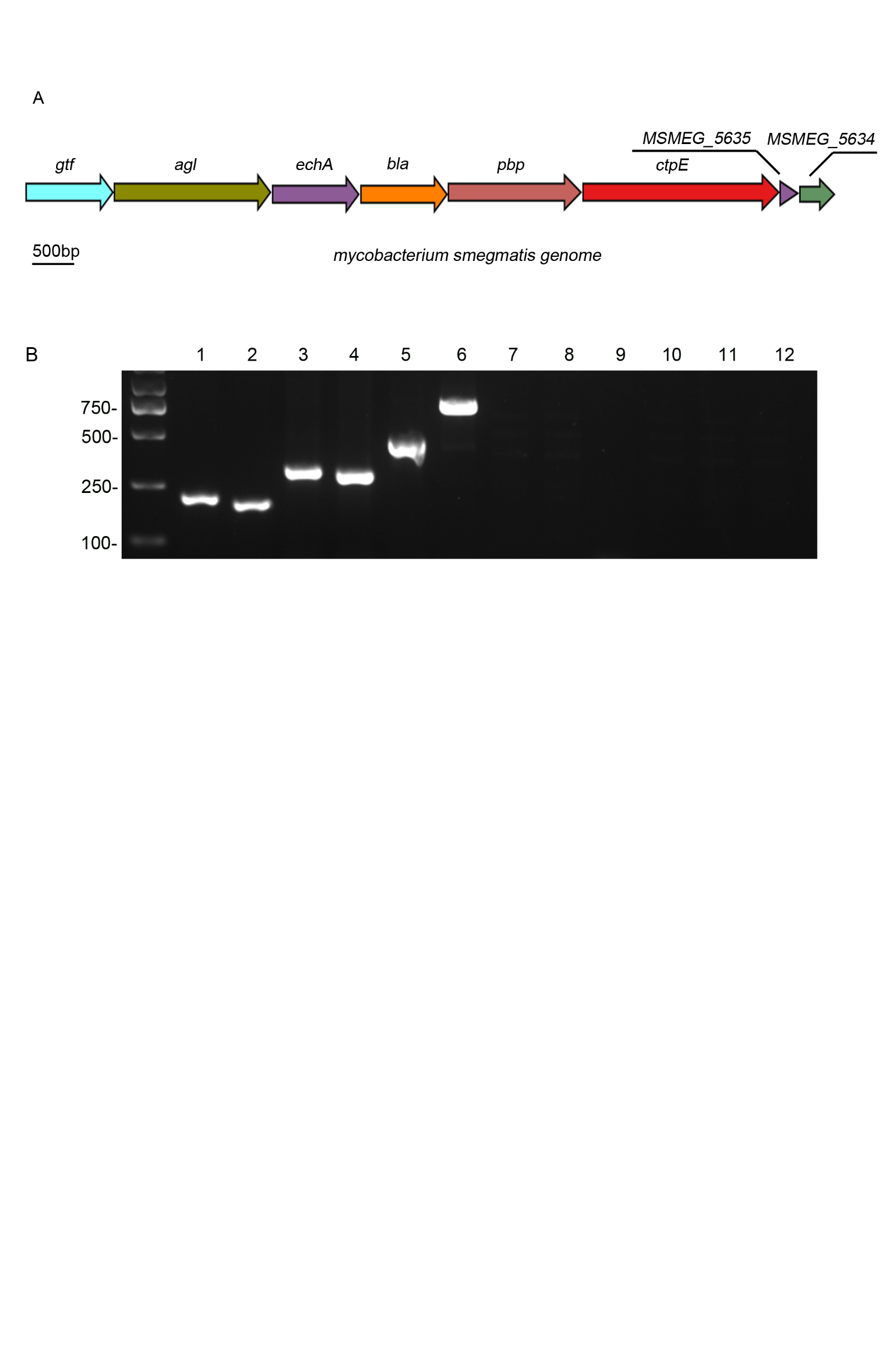


**Supplementary Figure 1.** (A) Schematic representation of the genetic organization of the *M. smegmatis* *MSMEG_5635/5634* operon. The genes and the proteins they encode are: *gtf*, glycosyltransferase; *agl*, amylo-α-1,6-glucosidase; *echA*, enoyl-CoA-hydratase; *bla*, metallo-β-lactamase; *pbp*, penicillin-binding protein 4; *ctpE*, cation-transporting P-type ATPase E. (B) RT-PCR analysis of *Mycobacterium smegmatis MSMEG_5635/5634* operon. Lane 1, amplified product of *MSMEG_5634*; lane 2, amplified product of *MSMEG_5635*; lane 3, amplified product of 16s RNA; lane 4, amplified product of *sigA*; The 16s RNA and *sigA* gene were used as controls. The intergenic regions between two genes were also ampliﬁed. Lane 5, ampliﬁed product of region between *MSMEG_5634* and *MSMEG_5635*; lane 6, ampliﬁed product of region between *MSMEG_5635* and *ctpE* (*MSMEG_5636*). Lanes 7-12, the RT negative controls corresponding to lanes 1-6 respectively with RNA samples untreated with reverse transcriptase.


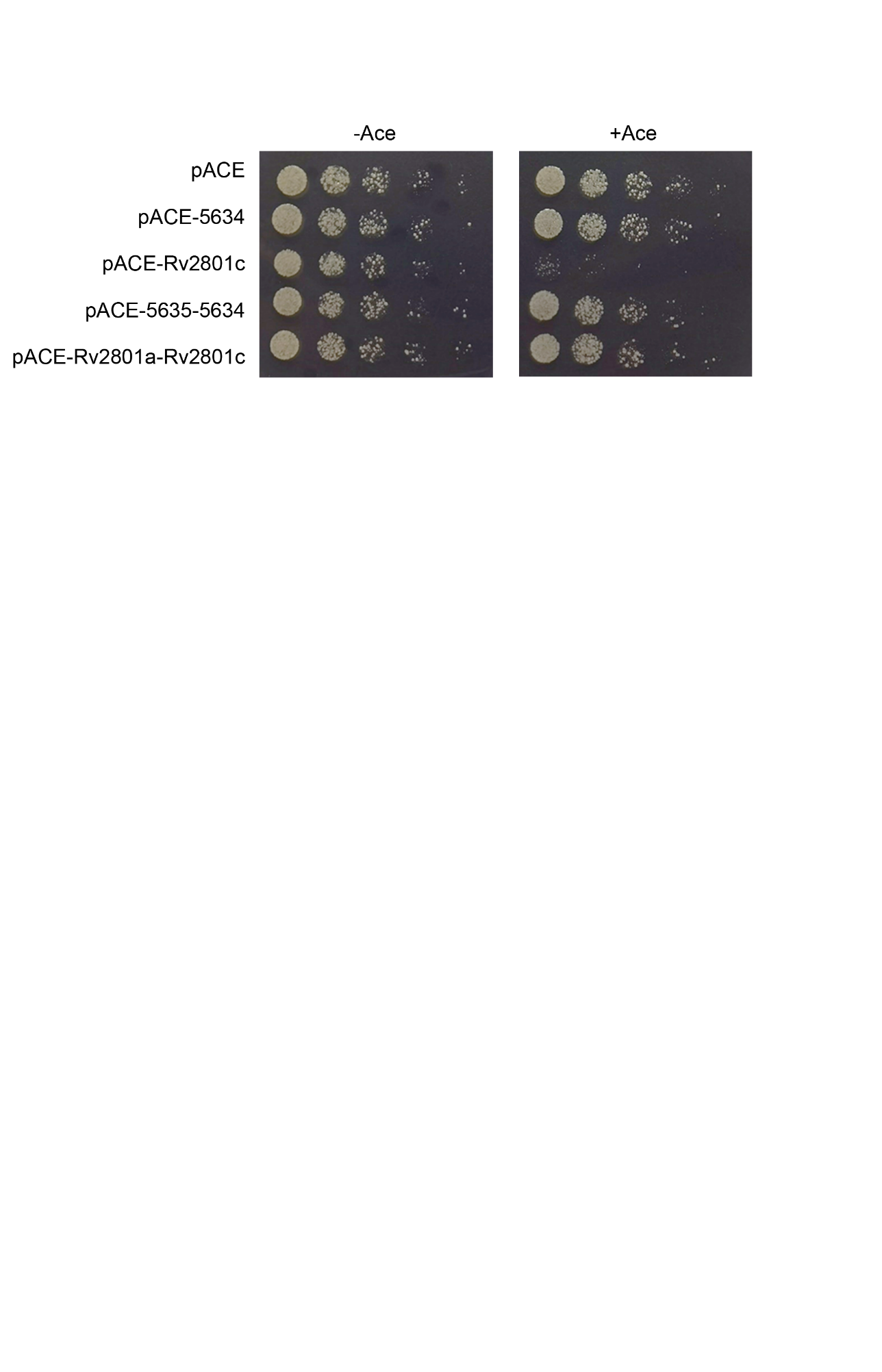


**Supplementary Figure 2.** *M. smegmatis* cells expressing MSMEG_5634 alone or MSMEG_5634-5635 under the control of the inducible acetamidase promoter in the pACE vector were serially diluted and plated on 7H10 solid medium including 0.2% Tween-80 with (right panel) or without (left panel) 0.2% acetamide as described previously ([Ramage et al., 2009](#_ENREF_2)). Growth was assessed after three days of incubation. *M. smegmatis* cells expressing Rv2801c alone or Rv2801a-Rv2801c with the pACE vector were used as controls.


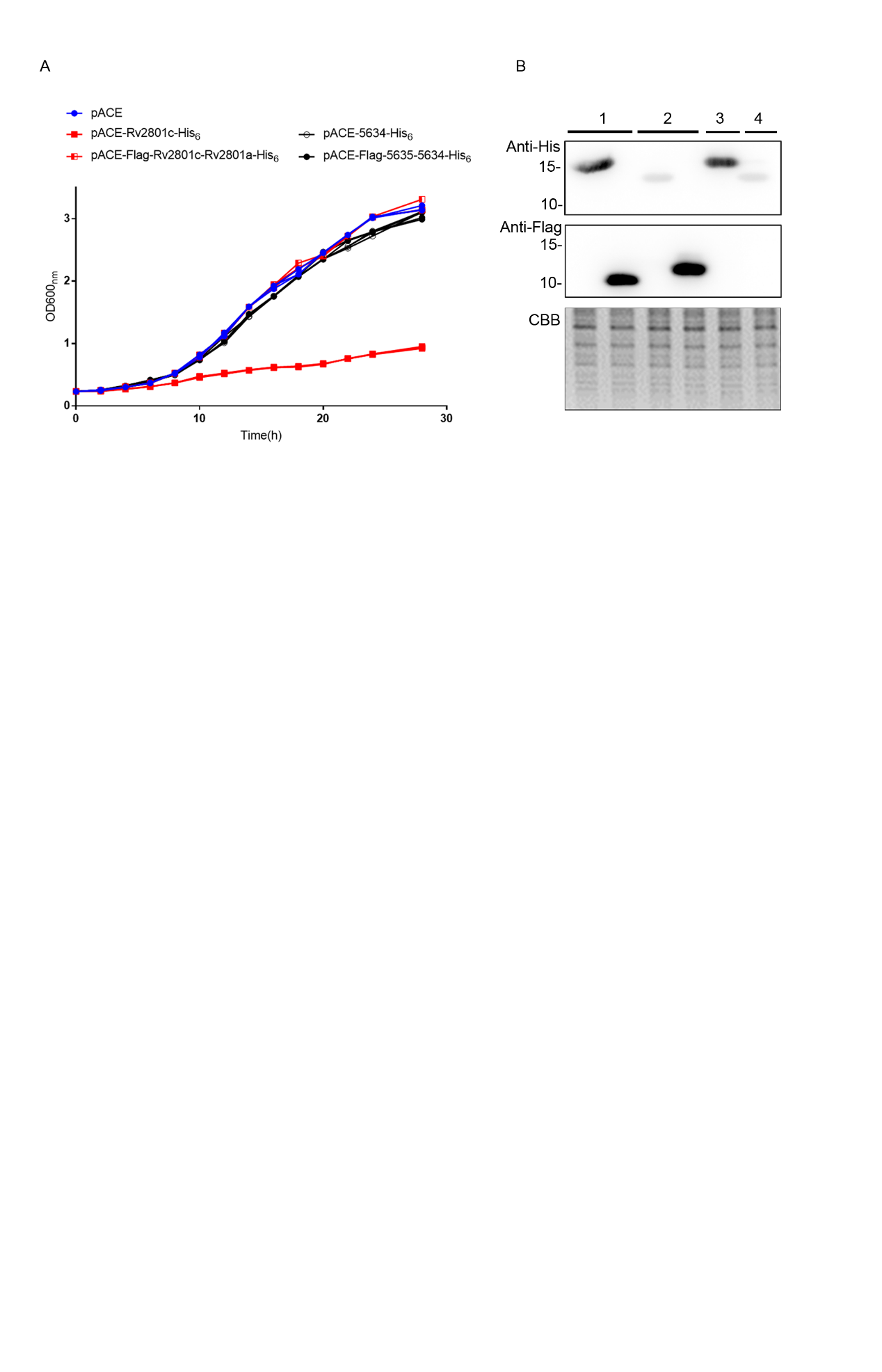


**Supplementary Figure 3.** *M. smegmatis* cells expressing Flag-MSMEG_5635 and MSMEG_5634-His_6_ under the control of the inducible acetamidase promoter in the pACE vector were cultured in 7H9 liquid medium with 0.2% acetamide. *M. smegmatis* cells harboring empty pACE vector and *M. smegmatis* cells expressing Flag-Rv2801a and Rv2801c-His_6_ with the pACE vector were used as controls. (A) The OD_600 nm_ of each *M. smegmatis* culture was measured every two hours. (B) The expression levels of MSMEG_5634-His_6_, Flag-MSMEG_5635, Rv2801c-His_6_, and Flag-Rv2801a in each strain were detected by Western blot. 1, *M. smegmatis* cells expressing Flag-MSMEG_5635 and MSMEG_5634-His_6_; 2, *M. smegmatis* cells expressing Flag-Rv2801a and Rv2801c-His_6_; 3, *M. smegmatis* cells expressing MSMEG_5634-His_6_; 3, *M. smegmatis* cells expressing Rv2801c-His_6_.

##
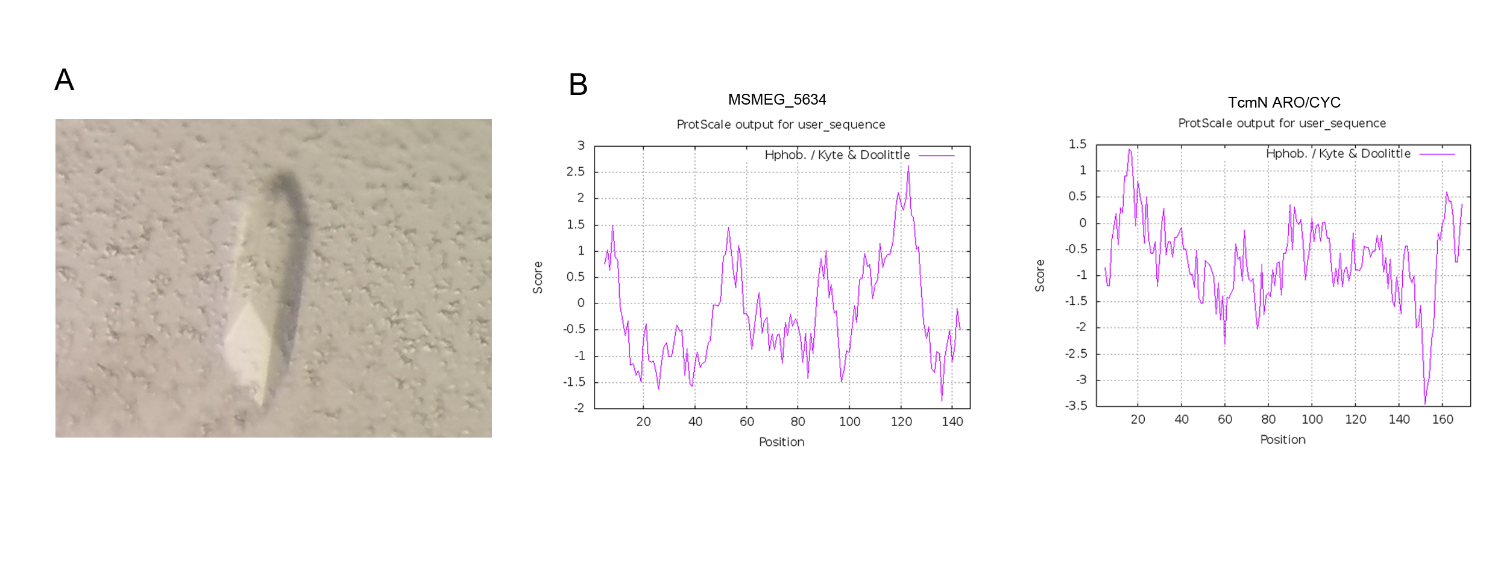


**Supplementary Figure 4**. (A) MSMEG_5634 protein crystal. (B) The hydrophobic regions of MSMEG_5634 and TcmN ARO/CYC analyzed by ExPASy ProtScale. A score with a positive value represents a hydrophobic region, and a score with a negative value represents a hydrophilic region.


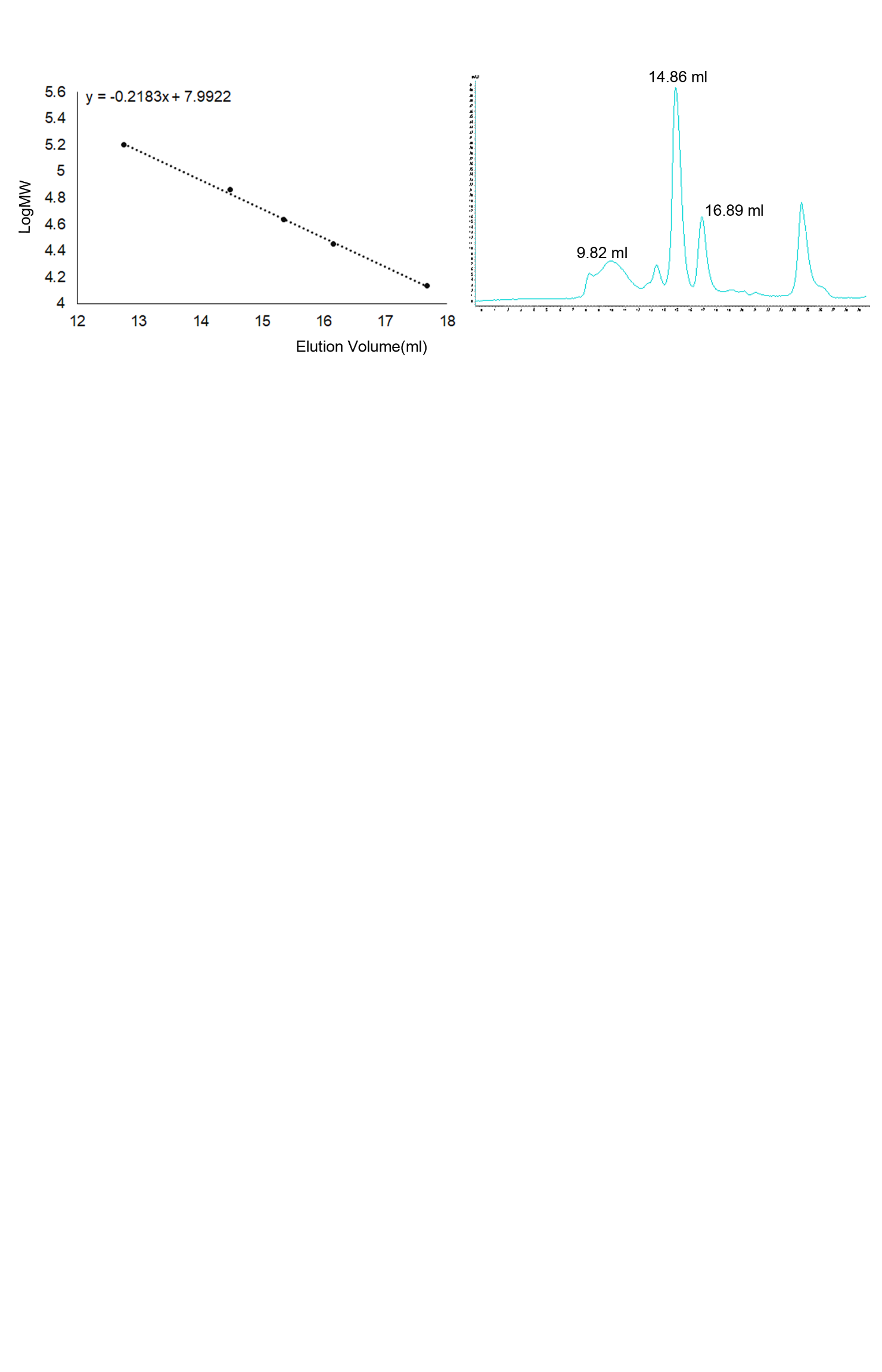


| Elution Volume (ml) | 9.82 | 14.86 | 16.89 |
| --- | --- | --- | --- |
| Theoretical MW (kDa) | 705.5 | 56.0 | 20.2 |

**Supplementary Figure 5.** A standard curve was obtained with Gel Filtration Molecular Weight Markers run on a Superdex 200 Increase 10/300 GL column. The Gel Filtration Molecular Weight Markers included Ribonuclease (13.7 kDa), Chymotrypsin (25 kDa), Ovalbumin (43 kDa), Albumin (66 kDa) and Aldolase (158 kDa) (left panel). The total protein sample copurified with MSMEG_5634-His_6_ expressed in *M. smegmatis* was subjected to size exclusion chromatography on a Superdex 200 Increase 10/300 GL column (right panel). The theoretical molecular weight of each fraction peak was calculated according to the standard curve.


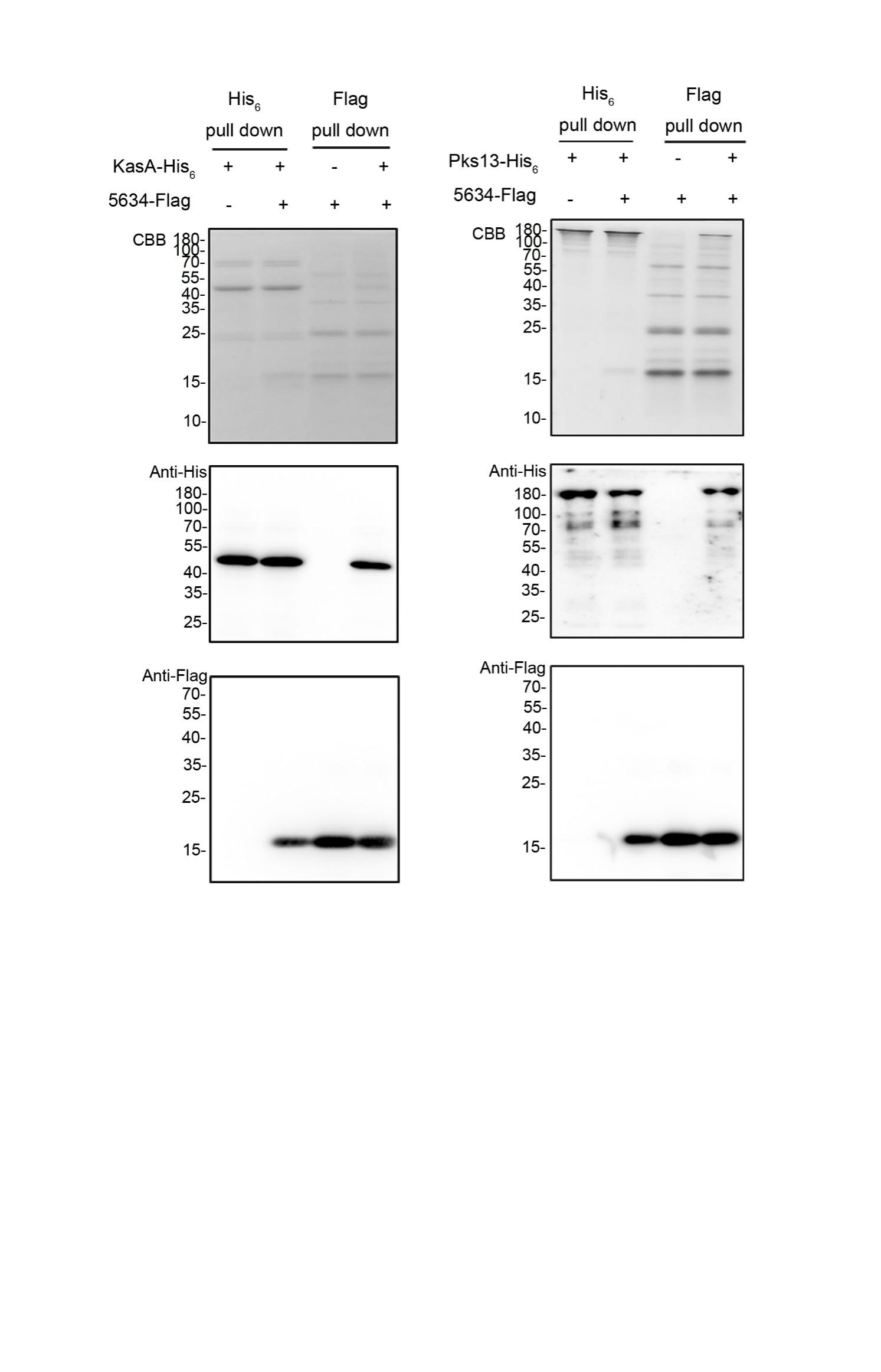


**Supplementary Figure 6.** The whole gels corresponding to the SDS-PAGE and Western blot analysis of Figure 3C.


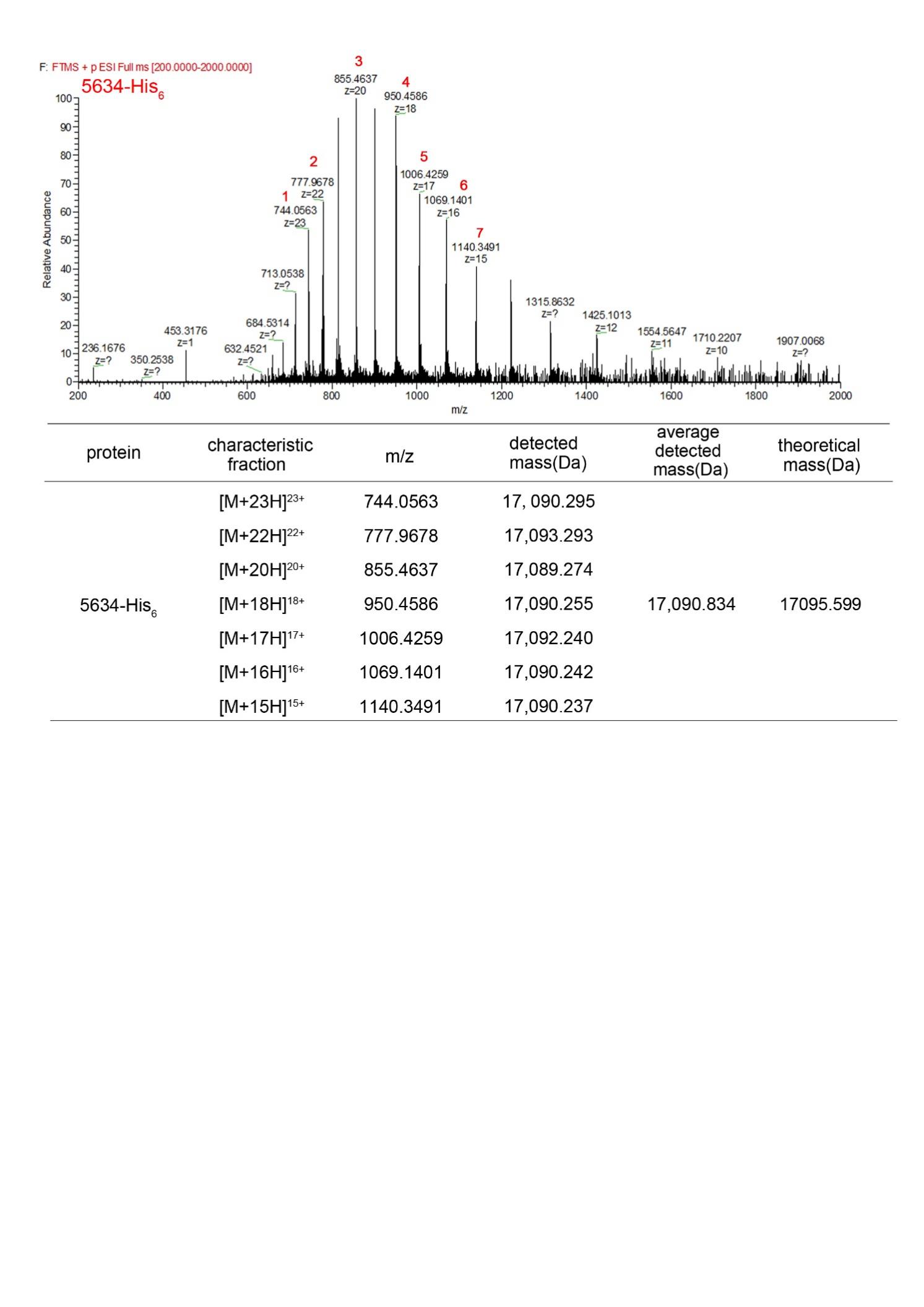
**Supplementary Figure 7.** Determination of the molecular mass of MSMEG_5634-His_6_ by UPLC-HR-MS. The detailed data are listed under the mass spectrometry results.


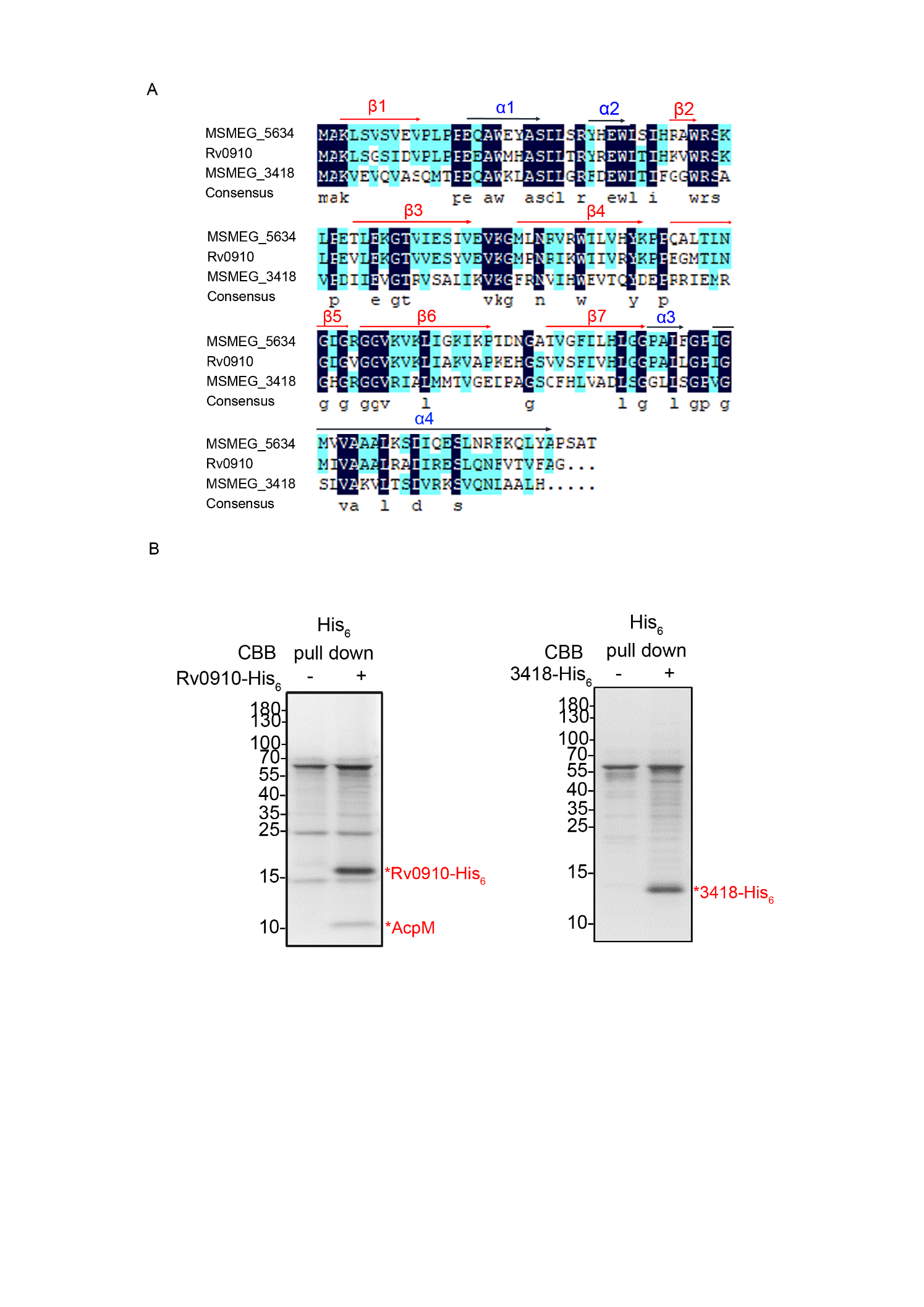


**Supplementary Figure 8.** **(A)** Alignment of MSMEG_5634 and its homologous proteins Rv0910 and MSMEG_3418. The secondary structure elements of MSMEG_5634 are shown at the top of the alignment. **(B)** Rv0910-His_6_ and MSMEG_3418-His_6_ were expressed in *M. smegmatis* and purified by Ni^2+^-NTA affinity chromatography. The eluted proteins were collected and analyzed by Tricine-SDS-PAGE


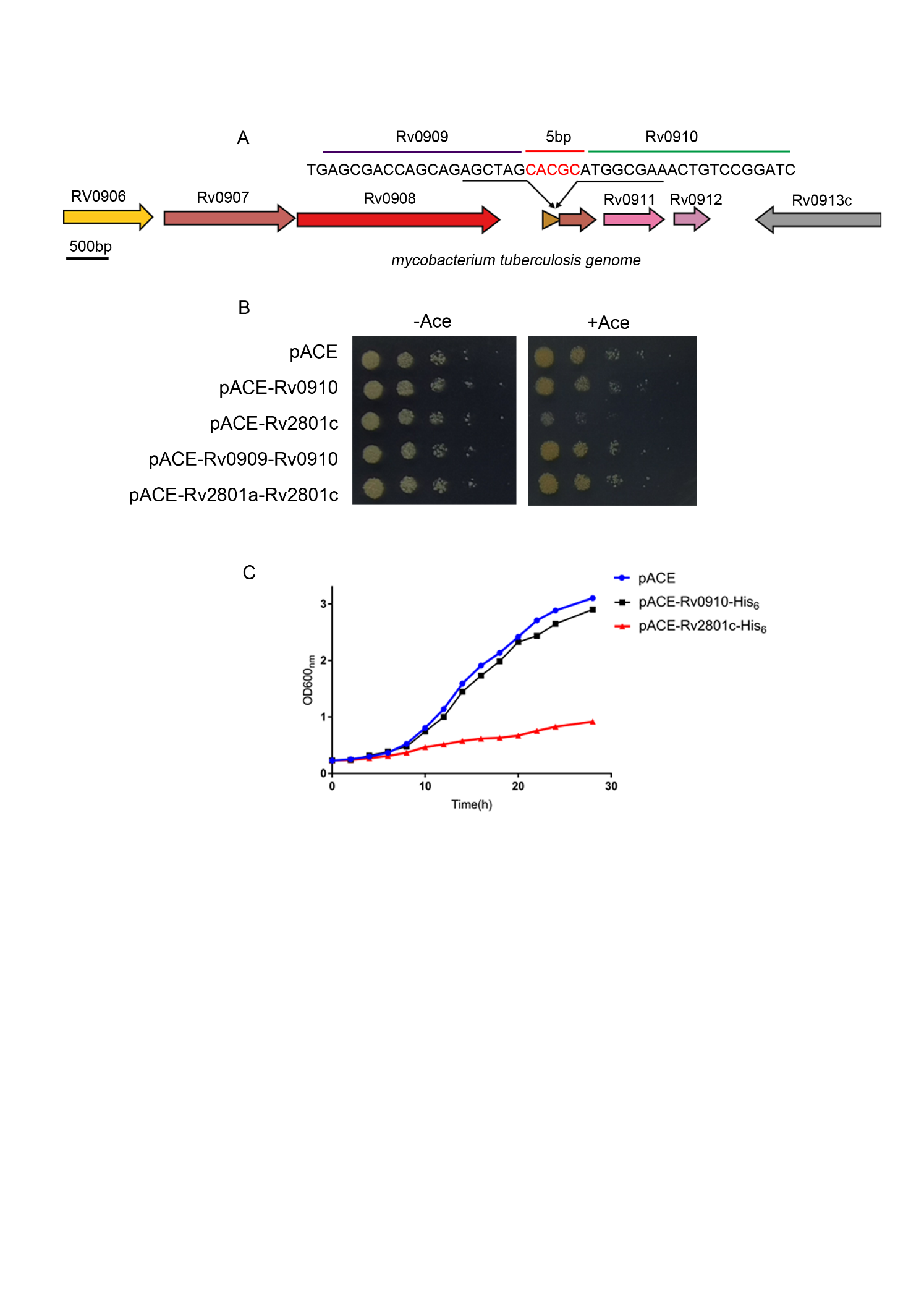


**Supplementary Figure 9**. Ectopic expression of Rv0910 does not inhibit the growth of *Mycobacterium smegmatis*. **(A)** Rv0910 gene locus in the genome of *Mycobacterium tuberculosis*. **(B)** *M. smegmatis* cells expressing Rv0910 alone or Rv0909-Rv0910 under the control of the inducible acetamidase promoter in the pACE vector were serially diluted and plated on 7H10 solid medium with (right panel) or without (left panel) 0.2% acetamide. **(C)** *M. smegmatis* cells expressing Rv0910 under the control of the inducible acetamidase promoter in the pACE vector were grown in 7H9 liquid medium. The OD_600 nm_ of the *M. smegmatis* culture was measured every two hours. Ectopic expression of Rv2801c in *M. smegmatis* under the control of the inducible acetamidase promoter in the pACE vector was used as a control.


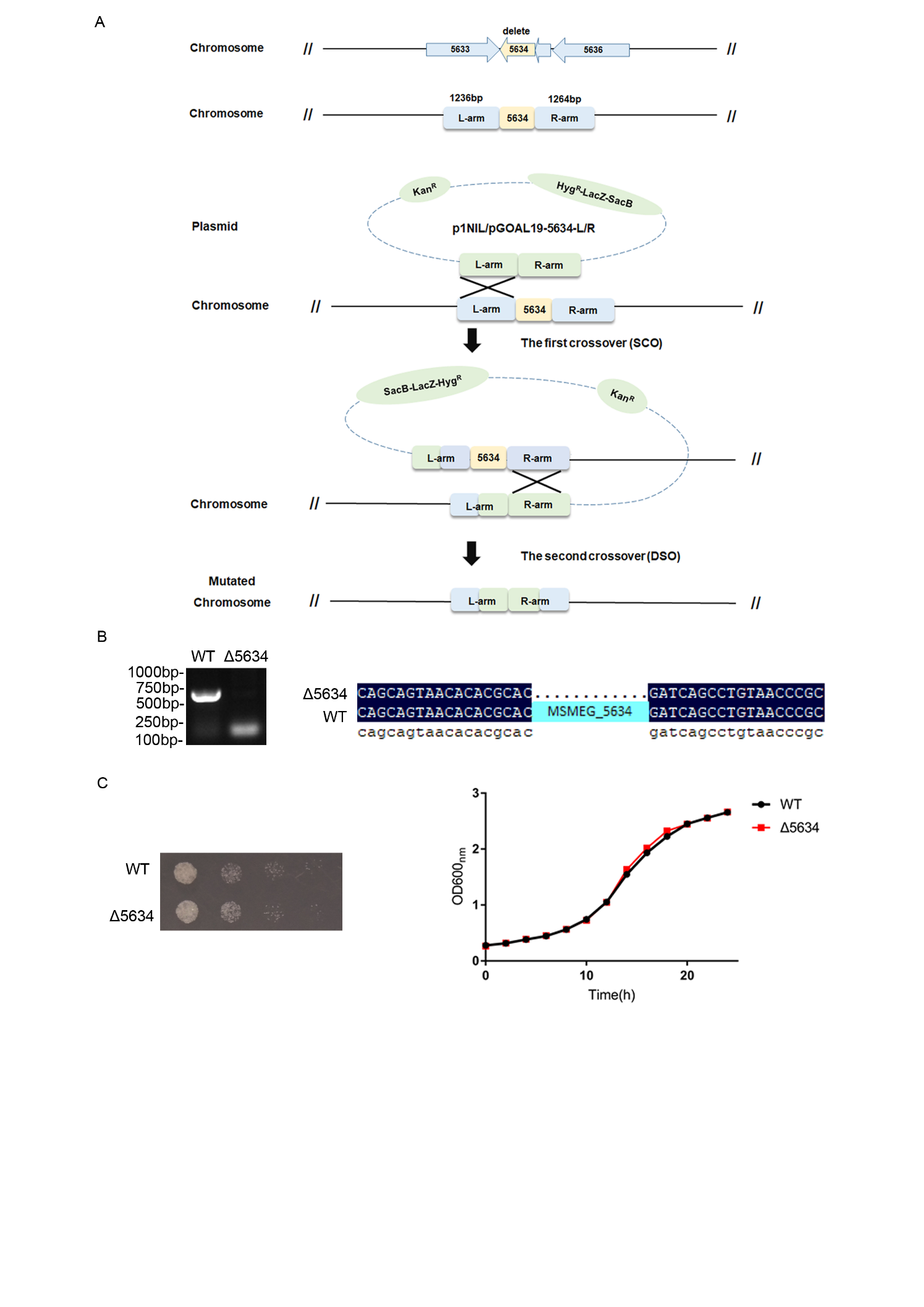


**Supplementary Figure 10.** **(A)** The *M. smegmatis* strain with *MSMEG_5634 gene* deletion were constructed by homologous recombination using the pNIL/pGOAL system. The details are described above in the Materials and Methods. **(B)** Deletion of the *MSMEG_5634* gene in *M. smegmatis* genomic DNA was identified by PCR and DNA sequencing. **(C)** Wild-type strain (WT) and the *MSMEG_5634* gene deletion strain (Δ5634) were cultured on 7H10 solid medium for 48 h (left panel) or in 7H9 liquid medium shown with the OD_600nm_ values measured every two hours (right panel).


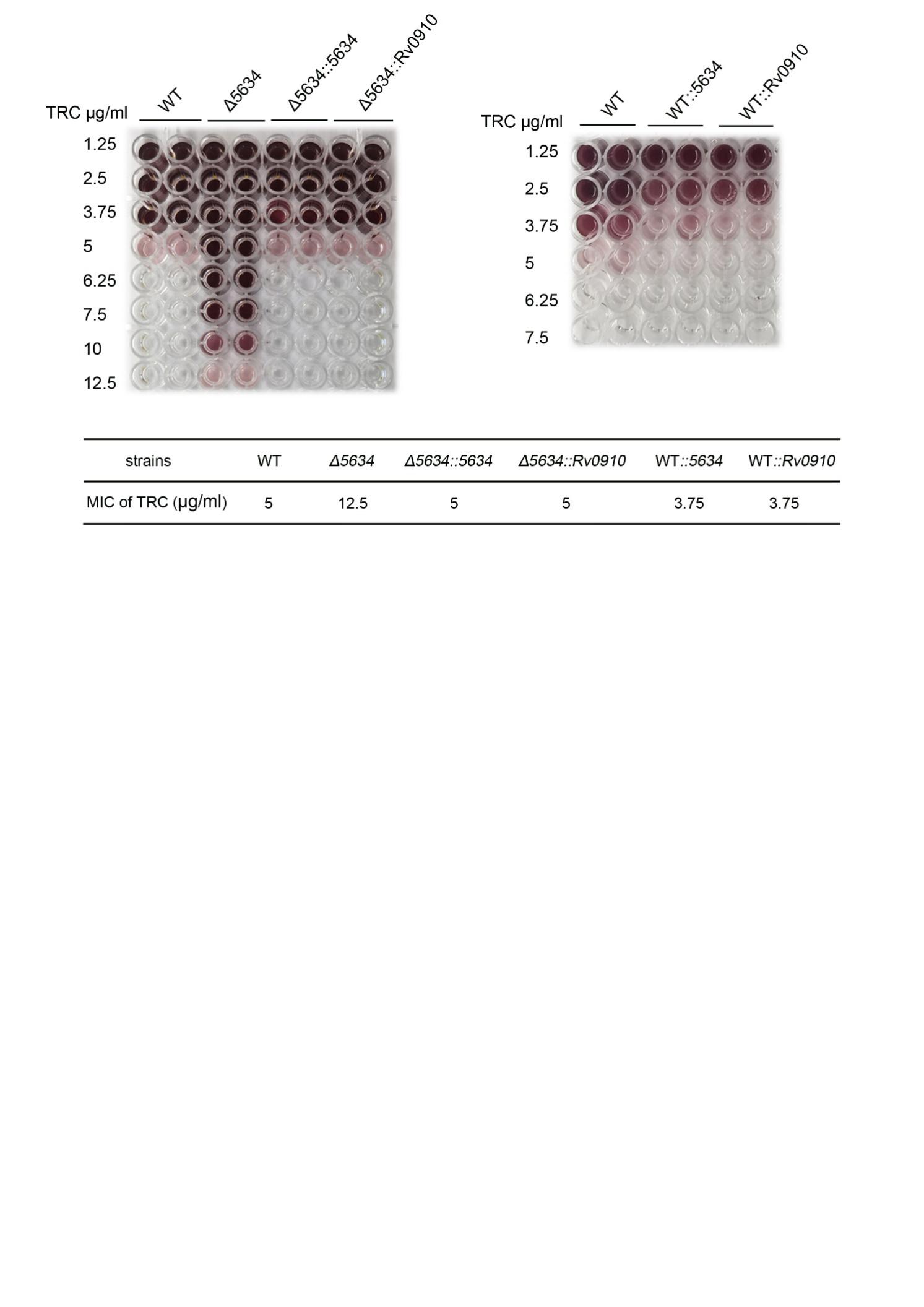


**Supplementary Figure 11.** The impacts of MSMEG_5634 on the minimum inhibitory concentration (MIC) of triclosan (TRC) in *M. smegmatis*. The indicated *M. smegmatis* strains (1×10^5^ cells/well) were cultured in 7H9 medium for 24 h at 37 °C under TRC treatment at different concentrations in the presence of acetamide (0.2%) with TTC (0.02%) as the indicator. WT, the wild-type cells containing empty pACE vector; *Δ5634*, the *MSMEG_5634*-deleted cells containing empty pACE vector; *Δ5634::5634* and *Δ5634::Rv0910* are the *MSMEG_5634*-deleted cells with the ectopic expression of MSMEG_5634 and Rv0910, respectively, under the control of the inducible acetamidase promoter in the pACE vector; WT*::5634* and WT*::Rv0910* are the wild-type cells with the ectopic expression of MSMEG_5634, and Rv0910, respectively, under the control of the inducible acetamidase promoter in the pACE vector.

## Supplementary Tables

**Table S1.** Data collection and refinement statistics of the MSMEG_5634 crystal

| MSMEG_5634 | |
| --- | --- |
| **Data collection** |  |
| beamline | SSRF-BL17U1 |
| Space group | *C*2 |
| Cell dimensions |  |
| a, b, c (Å) | 71.07, 55.01, 40.73 |
| γ α, β, γ (°) | 90.00, 112.75, 90.00 |
| Resolution (Å) | 50.00-1.90  (1.93-1.90)* |
| R_merge_ | 0.098 (0.596) |
| I / σI | 33.3 (8.5) |
| Completeness (%) | 98.3 (99.6) |
| Redundancy | 3.3 (3.4) |
| **Refinement** |  |
| Resolution (Å) | 22.19-1.90 |
| No. reflections | 21971 |
| R_work_ / R_free_ | 0.230/0.282 |
| No. atoms |  |
| Protein | 1121 |
| Water | 52 |
| B-factors (Å^2^) |  |
| Protein | 19.9 |
| Water | 26.8 |
| R.m.s. deviations |  |
| Bond lengths (Å) | 0.007 |
| Bond angles (°) | 1.018 |

**Table S2.** Identification of the proteins copurified with MSMEG_5634-His_6_ by trypsin digestion and MS peptide mapping

| **protein** | **coveragege** | **peptide map*** |
| --- | --- | --- |
| Pks13 | 37% | MTVNEMREWLRNWVANATGQSADAIDESTPMVELGLSSRDAVAMASDIEDLTGVTLTATVAFRHPTIESLATVIIEGEPEPEPYDEDEDWSRTRDVEDIAIVGVATRFPGDLNTPDEMWEALLEGKDCVTDLPEDRWTEFLDEPRIAERVKKARTRGGYLTDIKGFDSEFFALSKMEADNIDPQQRMALELTWEALEHARIPASSLRGESVGVYIGSSTNDYSFLAMSDPSIAHPYAITGTASSIIANRVSYFYDFRGPSVAVDTACSSSLVATHQGVQALRAGEADVAIVGGVNALVTPLVTVGFDEVGGVLAPDGRIKSFSSDADGYARSEGGGMLVLKRISDARRDGDQILAVIAGSAVNHDGRSNGLLAPNPDAQAEVLRKAYKDAGINPRDVDYIEAHGTGTILGDPIEADALGRIVGKGRPADKPALLGAVKSNLGHLESAAGAASLAKMTLALANDKLPPSINYAGPNPYIDFEKERLKVNDTVSDWPRYSGKAIAGVSGFGFGGANAHVVMREVLAGDLVEPEPEPEPEAKPEKSEADAVYVGGVRMDEYGEFIDEDEPAEGGDAYPSYDEDSYELPGITEAAQRLLEQAREELEAKEAEEPTKQLVPLAVSAFLTSRKRQAAAELADWIDSPEGRASSLESIGRSLSRRNHGRSRAVVLAHDHDEAIKGLRALAEGKQHPSVLSADGPVTNGPVWVLAGFGAQHRKMGKSLYLRNEVFAEWINKVDALIQDERGYSILELILDDNVDYTDATCEYPIEVVQLVIFAIQIALGELLRHHGAKPAAVVGQSLGEAAASYFAGGLSLADATRTICSRSHLMGEGEAMLFGEYIRLMALVEYSADEIKTVFSDYPDLEVCVYAAPTQTVIGGPPDQVDAIIARAESEGKFARKFQTKGASHTQQMDPLLGELAAELQGIEPKPLTTGYFSTVHEGTFIRPGSAPIHDVDYWKKGLRHSVYFTQGIRNAVDNGHTTFLELAPNPVALMQVGLTTASAGLHDAQLIATLARKQDEVESMISAMAQLYVHGHDLDFRTLFPRRSKGLAGALDFANIPPTRFKRKEHWLPAHFTGDSSAVMPGNHVATPDGRHVWEFVPRGKTDLAALVKAAAAQVLPDAKLAAFEQRAVPADNARLVTTLTRHPGGATVQVHARVEESFTLVYDAIVARANGAGVTALPVAVGAGVAVSGDVAGEGAGASVIEDDEPDAEILQDNLTAGAGMGADFQKWDPNSGETIGQRLGTIVGAAMGYEPEDLPWEVPLIELGLDSLMAVRIKNRVEYDFDLPPIQLTAVRDANLYNVEELIRYAIEHRDEVEQIAESQKGKTAEEIAAEQSELLGGASTVAELEAKLAEAGHPLAAKDSEDSENSEDNAAGAAAAAEASAVEGLEIPPPPTDPTGPGGAPIPPPPSDPSGPAQAASATDAPAGTVNKATAAAAAAKVLTQEAVTEALGADVPPRDAAERVTFATWAIVTGKSPGGIFNELPTVSEETAKKMAERLSERAEGTITVEDVLGAKTIEGLATIVREQLEEGVVDGFVRTLRPPKEGSNAVPLFVFHPAGGSTVVYEPLMKRLPADVPVYGLERVEGSIEERAAEYVPKLLEMHKGPFVLAGWSLGGALAYACAIGLKQSGADVRFVGLIDTVLPGEPIDQSKEGMRARWDRYARFAERTFNVEIPAIPYEELEKLDDEGQVKYVLEIVKESGVQIPGGIIEHQRTSYLDNRALDTVDIKPYDGHVTLYMADRYHDDAIVFEPAYATRKPDGGWGSFVSDLEVVHIGGEHIQAIDEPYIAKVGAHMSEALNRIEAQASKEDGAK |
| KasA | 46.8% | MTRPSTANGGYPNVVVTAVTATTSIAPDIESTWKGLLAGESGIRVLEDEFVTKWDLPVRIGGHLVDNIDDHMTRLDMRRMSYVQRMSKFLSKQLWENAGAPEVDPDRFAVVIGTGLGGGEKIVETYDAMNEGGPRKVSPLAVQMIMPNGAAAVVGLELGARAGVITPVSACSSGSEAIAHAWRQIVMGDADFAVCGGVEGGIEALPIAAFSMMRAMSTRNDDPQGASRPFDKDRDGFVFGEAGAMMIIETEEHAKARGAKPLARLMGAGITSDAFHMVAPAADGLRAGQAMKRAMETAGLDPKDIDHVNAHATATPIGDTAEANALRVAGVEHAAVYAPKSALGHSIGAVGALESILTVLALRDGVIPPTLNYETPDPEIDLDIVAGEPRYGEYKYAINNSFGFGGHNVALAFGRY |
| AcpM | 23% | MAATQEEIIAGLAEIIEEVTGIEPSEVTPEKSFVDDLDIDSLSMVEIAVQTEDKYGVKIPDEDLAGLRTVGDVVAYIQKLEEENPEAAAALREKFAADQ |
| GroL2 (CH60 2) | 62.4% | MSKQIEFNETARRAMEAGVDKLADAVKVTLGPRGRHVVLAKSFGGPQVTNDGVTIAREIDLEDPYENLGAQLVKSVATKTNDVAGDGTTTATVLAQALVRAGLRNVAAGANPIALGSGISKAADAVSEALLASATPVDDKKAIAQVATVSSRDEQVGELVGEAMTKVGHDGVVTVEESSTLETYLEVTEGVGFDKGFLSAYFVTDFDSQEAVLEDALVLLHRDKISSLPDLLPLLEKVAEAGKPLLIVAEDVEGEALSTLVVNAIRKTLKAVAVKAPFFGDRRKAFLDDLAIVTGGQVVNPDVGLLLREVGLEVLGSARRVVVNKDSTVIVDGGGTAEAIADRVKQIKSEIETTDSDWDREKLQERLAKLAGGVAVIKVGAATETDLKKRKEAVEDAVAAAKAAVEEGIVTGGGAALVQARSAVEKLRGELSGDEALGVDVFASALSAPLYWIATNAGLDGSVVVNKVSELPKGQGFNAATLEFGDLVSAGVVDPAKVTRSAVLNAASVARMILTTETAVVDKPADEDEHGHGHHHGHAH |

*The amino acid sequences of AcpM, Pks13, KasA and GroL2 are shown. The peptides shown in red were detected by the MS peptide mapping.

**Table S3.** The FAS-Ⅱ components identified by mass spectrometry analysis in the high molecular weight multiprotein fraction from the size exclusion chromatography of proteins coprecipitated with MSMEG_5634-His_6_

| Accession | Description | Score | Coverage | Unique Peptides |
| --- | --- | --- | --- | --- |
| A0R617 | Polyketide synthase OS=Mycobacterium smegmatis (strain ATCC 700084 / mc (2)155) GN=MSMEG_6392(PKS13) PE=4 SV=1 - [A0R617_MYCS2] | 336.61 | 37.67 | 60 |
| A0R3Y0 | Uncharacterized protein OS=Mycobacterium smegmatis (strain ATCC 700084 / mc (2)155) GN=MSMEG_5634 PE=4 SV=1 - [A0R3Y0_MYCS2] | 201.36 | 46.94 | 9 |
| A0R0B4 | 3-oxoacyl-[acyl-carrier-protein] synthase 1 OS=Mycobacterium smegmatis (strain ATCC 700084 / mc (2)155) GN=MSMEG_4327(KasA) PE=3 SV=1 - [A0R0B4_MYCS2] | 155.03 | 51.92 | 17 |
| A0R0B3 | Meromycolate extension acyl carrier protein OS=Mycobacterium smegmatis (strain ATCC 700084 / mc (2)155) GN=acpM PE=1 SV=1 - [ACPM_MYCS2] | 54.24 | 34.34 | 4 |
| P42829 | Enoyl-[acyl-carrier-protein] reductase [NADH] OS=Mycobacterium smegmatis (strain ATCC 700084 / mc (2)155) GN=inhA PE=1 SV=1 - [INHA_MYCS2] | 17.86 | 15.99 | 4 |

# References

Meniche, X., Otten, R., Siegrist, M.S., Baer, C.E., Murphy, K.C., Bertozzi, C.R., and Sassetti, C.M. (2014). Subpolar addition of new cell wall is directed by DivIVA in mycobacteria. *Proc Natl Acad Sci U S A* 111**,** E3243-3251.

Ramage, H.R., Connolly, L.E., and Cox, J.S. (2009). Comprehensive functional analysis of Mycobacterium tuberculosis toxin-antitoxin systems: implications for pathogenesis, stress responses, and evolution. *PLoS Genet* 5**,** e1000767.

Voskuil, M.I., Schnappinger, D., Visconti, K.C., Harrell, M.I., Dolganov, G.M., Sherman, D.R., and Schoolnik, G.K. (2003). Inhibition of respiration by nitric oxide induces a Mycobacterium tuberculosis dormancy program. *J Exp Med* 198**,** 705-713.
